# Multifactorial Origins of Heart and Gut Defects in *nipbl*-Deficient Zebrafish, a Model of Cornelia de Lange Syndrome

**DOI:** 10.1371/journal.pbio.1001181

**Published:** 2011-10-25

**Authors:** Akihiko Muto, Anne L. Calof, Arthur D. Lander, Thomas F. Schilling

**Affiliations:** 1 Department of Developmental and Cell Biology, University of California, Irvine, California, United States of America; 2 Center for Complex Biological Systems, University of California, Irvine, California, United States of America; 3 Department of Anatomy and Neurobiology, University of California, Irvine, California, United States of America; The Wellcome Trust Sanger Institute, United Kingdom

## Abstract

Cornelia de Lange Syndrome (CdLS) is the founding member of a class of multi-organ system birth defect syndromes termed cohesinopathies, named for the chromatin-associated protein complex cohesin, which mediates sister chromatid cohesion. Most cases of CdLS are caused by haploinsufficiency for Nipped-B-like (Nipbl), a highly conserved protein that facilitates cohesin loading. Consistent with recent evidence implicating cohesin and Nipbl in transcriptional regulation, both CdLS cell lines and tissues of *Nipbl*-deficient mice show changes in the expression of hundreds of genes. Nearly all such changes are modest, however—usually less than 1.5-fold—raising the intriguing possibility that, in CdLS, severe developmental defects result from the collective action of many otherwise innocuous perturbations. As a step toward testing this hypothesis, we developed a model of *nipbl*-deficiency in zebrafish, an organism in which we can quantitatively investigate the combinatorial effects of gene expression changes. After characterizing the structure and embryonic expression of the two zebrafish *nipbl* genes, we showed that morpholino knockdown of these genes produces a spectrum of specific heart and gut/visceral organ defects with similarities to those in CdLS. Analysis of *nipbl* morphants further revealed that, as early as gastrulation, expression of genes involved in endodermal differentiation (*sox32*, *sox17*, *foxa2*, and *gata5*) and left-right patterning (*spaw*, *lefty2*, and *dnah9*) is altered. Experimental manipulation of the levels of several such genes—using RNA injection or morpholino knockdown—implicated both additive and synergistic interactions in causing observed developmental defects. These findings support the view that birth defects in CdLS arise from collective effects of quantitative changes in gene expression. Interestingly, both the phenotypes and gene expression changes in *nipbl* morphants differed from those in mutants or morphants for genes encoding cohesin subunits, suggesting that the transcriptional functions of Nipbl cannot be ascribed simply to its role in cohesin loading.

## Abstract 2

*nipbl*-deficient zebrafish provide evidence that heart and gut defects in Cornelia de Lange Syndrome are caused by combined effects of multiple gene expression changes that occur during early embryonic development.

## Author Summary

Although best known for its role in chromatid cohesion, cohesin is increasingly seen as a regulator of gene expression. In Cornelia de Lange Syndrome (CdLS), partial deficiency for *NIPBL*, which encodes a cohesin regulator, is associated with small changes in the expression of many genes (similar effects are seen in Nipbl-deficient mice and flies). Are such changes responsible for pervasive developmental defects in CdLS? To address this, we used morpholino oligonucleotides to quantitatively reduce levels of Nipbl protein and Nipbl target genes in zebrafish embryos. Combined knockdown of both zebrafish *nipbl* genes produced heart and gut defects with similarities to those observed in CdLS. Nipbl-deficient embryos showed quantitative changes in the expression of several genes involved in the specification of endoderm, which both gives rise to gut and provides a substrate for cardiac precursor migration, as well as genes that regulate left-right asymmetry. Functional studies of these putative targets suggest that changes in their expression collectively, and in some cases synergistically, contribute to the observed phenotypes. These findings suggest that birth defects in CdLS result from combinatorial, quantitative effects of NIPBL on gene expression, and suggest that cardiac and visceral organ defects in CdLS arise during early embryonic development.

## Introduction

Cohesin is a multi-protein complex that associates with the chromosomes of all eukaryotic cells, and mediates sister chromatid cohesion, ensuring appropriate segregation of chromosomes during cell division [1],[2]. Recent work suggests that cohesin also acts during interphase to regulate gene expression [3]–[5]. Studies on a set of human birth defect syndromes recently termed “cohesinopathies,” along with work in *Drosophila* and in cell culture, point to roles for cohesin in various processes such as long-range promoter/enhancer communication, insulator action, and gene activation in the presence of polycomb silencing activity (reviewed by [1]).

Many of these studies have focused on a protein known variously as Scc2 (in yeast and *Xenopus*), Nipped-B (in *Drosophila*), or Nipped-B-like (Nipbl, in most vertebrates), which is not itself a cohesin subunit, but is required for the loading of cohesin onto DNA [6]–[9]. Haploinsufficiency for *NIPBL* is the most frequent cause of Cornelia de Lange Syndrome (CdLS) (OMIM #122470), the most common of the cohesinopathies [10]–[13]. CdLS is a multi-organ birth defects syndrome characterized by low birth weight, short stature, and variably penetrant structural abnormalities of the skeleton, heart, gut, kidney, genitalia, eyes, and teeth, together with abnormalities in cognition and behavior [14]–[20]. A recently developed mouse model of *Nipbl* haploinsufficiency displays many of these abnormalities, along with nearly 80% perinatal mortality [21]. Interestingly, in both man and mouse, *Nipbl* heterozygotes show only a ∼30% reduction in *Nipbl* mRNA and protein [21]–[23], presumably a result of autoregulation of the wild-type allele. This implies an extraordinary sensitivity of developmental events to small changes in the levels of this molecule. Indeed, clinical data suggest that a mere 15% decrease in *NIPBL* levels produces a recognizable phenotype [22].

The fact that such small changes in the levels of *NIPBL/Nipbl/Nipped-B* expression have little or no effect on chromosome cohesion [21],[24]–[26] has led to the hypothesis that developmental abnormalities in CdLS are the result of dysregulated gene expression. Human and mouse studies indicate that hundreds of genes are expressed abnormally in *NIPBL/Nipbl/Nipped-B* heterozygotes in any given cell type or organ [21],[23]. Yet with few exceptions, the changes in gene expression are modest, nearly always less than 2-fold, and typically less than 1.5-fold. Similarly small changes in gene expression are seen in *Drosophila* and mammalian cell lines when Nipped-B/NIPBL expression is knocked down using RNAi [5],[27]. Are such small effects, perhaps collectively, the cause of pervasive developmental abnormalities? Or are there some much larger effects of NIPBL deficiency on the expression of critical developmental genes (cf. [27]) that have just not yet been detected (e.g., because such genes are expressed at early stages, for limited time periods, or in limited groups of cells)?

Answering this question is critical for more than just an understanding of cohesinopathies and cohesin function: the structural abnormalities in CdLS include some of the most common, clinically significant, isolated (non-syndromic) birth defects in humans, such as abnormalities of cardiac septum development [15]–[17],[28]. If such common defects can be reliably caused by the collective actions of many small changes in gene expression, it suggests a model for birth defects very different from the single-gene-centered models that are supported by much of the experimental literature.

To address this question definitively, one must be able to manipulate Nipbl or cohesin levels and quantitatively monitor phenotypic and gene expression changes from the earliest stages of development. Here we achieve this goal in the zebrafish, employing morpholino (MO)-mediated knockdown of Nipbl and several of its putative targets. We find that Nipbl knockdown produces heart, gut, and laterality defects with similarities to those seen in CdLS. Analysis of gene expression suggests that early, modest changes in expression of key regulatory genes involved in endodermal differentiation, migration, and left-right (L/R) patterning are likely to be the primary causes of such defects. Quantitative analyses of the expression and functional knockdown of two endodermal determinants, *sox17* and *foxa2*, supports the idea that developmental defects in CdLS can arise from the synergistic effects of changes in expression of Nipbl target genes.

## Results

### Two Zebrafish *nipbl* Genes

**Figure 1:**
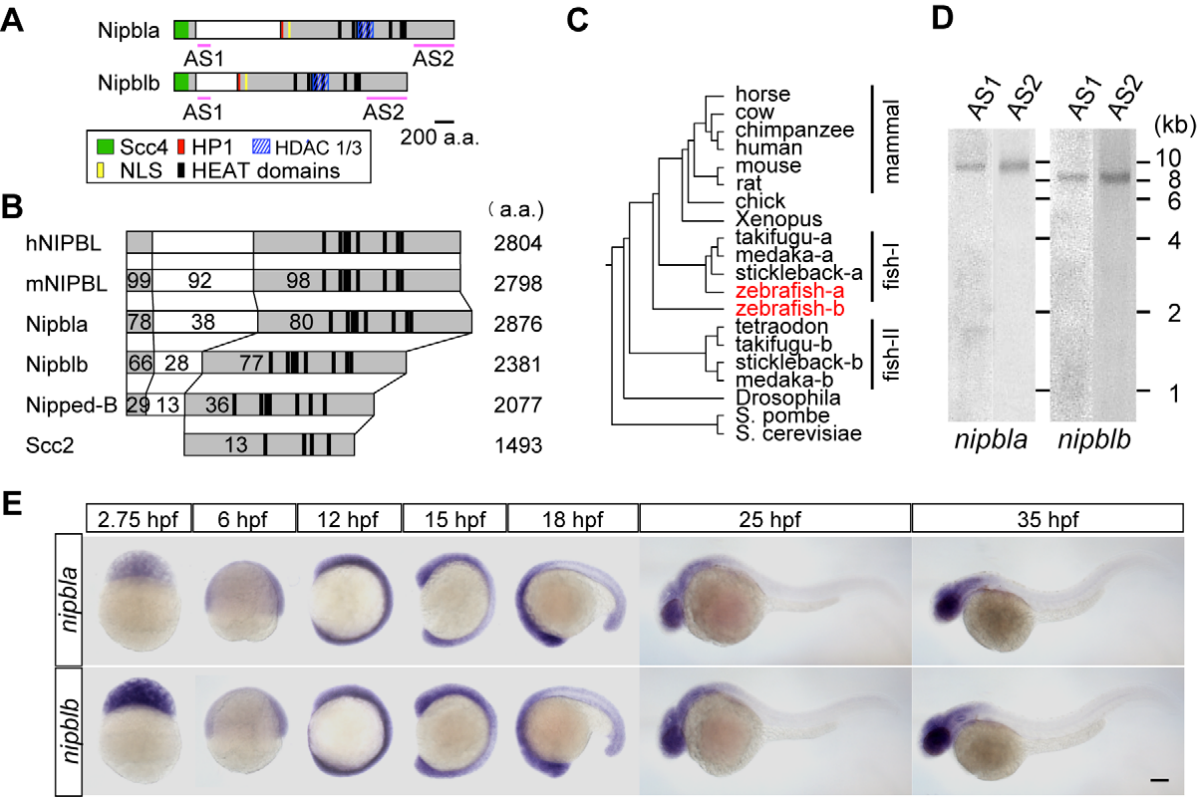
Zebrafish *nipbl* genes. (A) Domain structure of Nipbla and Nipblb proteins. N- and C-terminal conserved regions are shown in gray; predicted domains and motifs as colored boxes. Pink lines indicate positions used for antisense RNA probes. (B) Comparison of Nipbl orthologs among species. Numbers indicate amino acid identity to human NIPBL, as determined by Clustal W analysis (http://clustalw.ddbj.nig.ac.jp/top-j.html). (C) Phylogenic tree of Nipbl proteins, constructed by the NJ method (http://www.ddbj.nig.ac.jp/), using full-length amino acid sequences from the Ensembl database. (D) Expression of *nipbla* and *nipblb* mRNAs at 9 hpf was analyzed by Northern blotting (10 µg total RNA per lane) using DIG-labeled antisense RNA probes. Two different probes for each gene (see panel A) were used. (E) Expression patterns analyzed by ISH with *nipbla* (upper) and *nipblb* (lower) AS1 probes at indicated stages. Views are lateral, with anterior to the top (2.75–15 hpf) and left (18–35 hpf). Scale bar: 100 µm.

BLAST searches using the amino acid sequence of human NIPBL identified two zebrafish genes on chromosomes 10 and 5, referred to as *nipbla* and *nipblb*, respectively. We cloned full-length cDNAs for both from wild-type zebrafish (AB strain) by RT-PCR and 5′-RACE. The *nipbla* and *nibplb* cDNAs contained long open-reading frames encoding proteins of 2,876 and 2,381 amino acids in length, respectively (Figures 1A, S1). Predicted amino acid sequences of Nipbla and Nipblb are 70% identical to each other and 66% identical to that of human NIPBL. In both proteins, highly conserved N-terminal and C-terminal regions (∼200 and ∼1,700 amino acids, respectively) flank a less-conserved central region that is much shorter in Nipblb than in Nipbla (Figures 1A,B, S1). Conserved HEAT domains, a putative nuclear localization signal (NLS), and several protein-binding motifs (for SCC4 [9]; HDAC1/3 [29]; and HP-1 [30]), are also found in both proteins (Figures 1A, S1).

Alignment with genome sequences revealed that *nipbla* and *nipblb* contain 47 and 45 exons, respectively, and in each case the initial ATG codon is located in exon 2. Exon-intron structure resembles mammalian orthologs, though *nipblb* lacks one exon found in *nipbla* and other orthologs, as well as the intron that separates exons 36 and 37. Both *nipbl* genes lie in genomic neighborhoods syntenic to that surrounding human NIPBL: zebrafish orthologs of human *SLC1A3* (which encodes a high-affinity glutamate transporter), *LOC556181*, and *slc1a3a*, lie upstream of *nipbla* and *nipblb* on chromosomes 10 and 5, respectively. Other distantly related teleosts such as medaka, Fugu, and stickleback also have two *nipbl* genes, though one is much more similar to the two zebrafish Nipbls (Figure 1C), raising the possibility that both zebrafish genes arose from a single teleost duplicate.

Northern blot analysis revealed *nipbla* and *nipblb* transcripts of approximately 10 and 8.5 kb, respectively (Figure 1D). Both are detected in the early blastula, 2.5 h post fertilization (hpf, 256-cell), before the onset of zygotic gene expression, and expression progressively increases, reaching a peak at late gastrula stages (9 hpf, 90% epiboly), before decreasing by 26 hpf. While *NIPBL* mRNAs of multiple sizes have been reported in human tissues [11],[13], transcripts of both zebrafish genes were detected as single bands at all stages examined; this was confirmed using two different probes against 5′- and 3′-ends (Figures 1D, S2). In situ hybridization (ISH) revealed that both *nipbla* and *nipblb* are expressed in similar spatiotemporal patterns (Figure 1E). Maternal transcripts of both genes were detected throughout the blastoderm, although staining was stronger for *nipblb* (consistent with the Northern blotting results). Ubiquitous expression continues until early somitogenesis (12 hpf), after which transcript levels gradually decrease in the trunk (15–18 hpf), with strong expression becoming restricted to the head by 25 hpf (Figure 1E). These expression patterns are similar to those of cohesin subunits, *smc3* [31] and *rad21* [32],[33].

### Requirements for *nipbl* Genes in Heart and Visceral Organ Development

**Figure 2:**
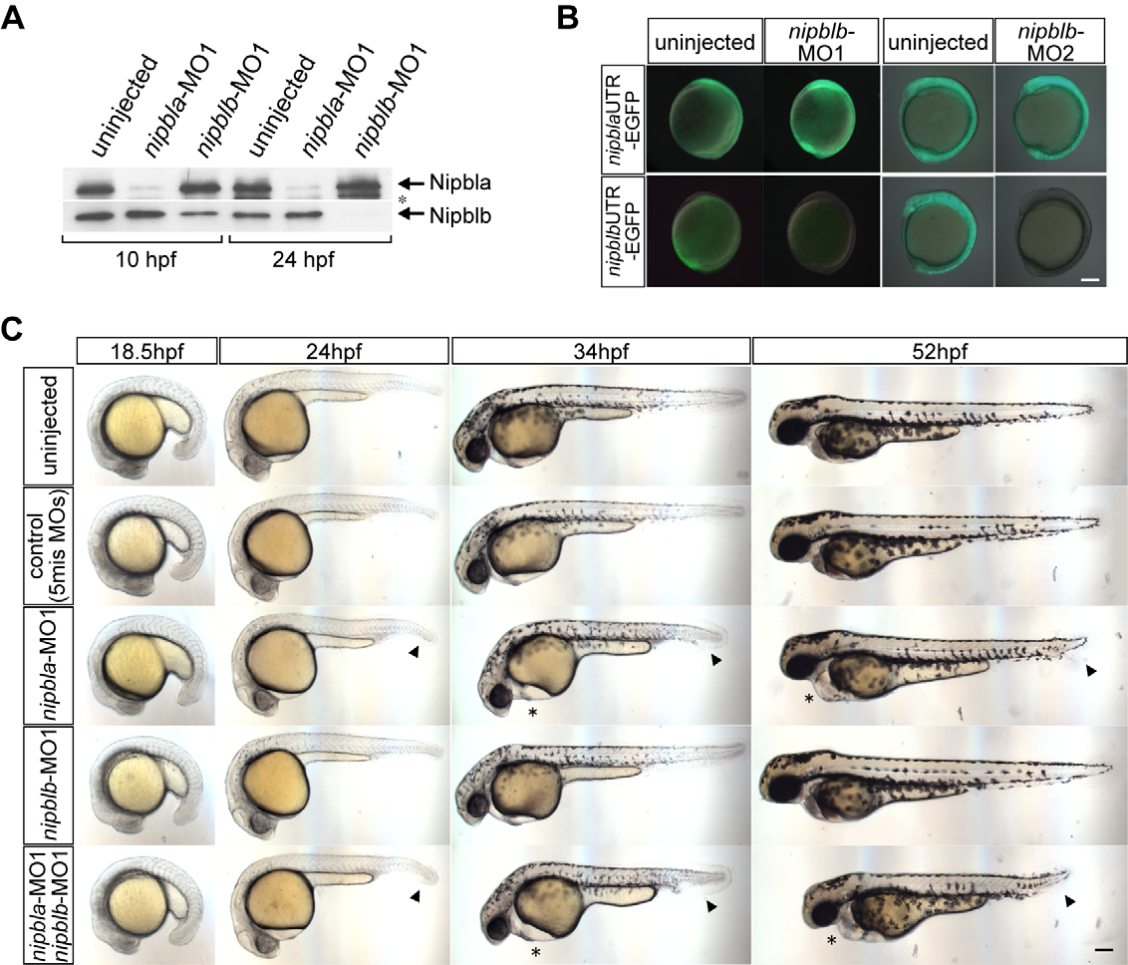
Morpholino knock-down of Nipbls. (A) Total protein lysates (10 embryos per lane) from uninjected embryos or embryos injected with either 0.5 ng of *nipbla*-MO1 or 0.5 ng of *nipblb*-MO1 were prepared at 10 and 24 hpf and subjected to Western blotting with anti-Nipbla and Nipblb-antibodies. Positions of Nipbla and Nipblb proteins are shown on the right. A lower band recognized by anti-Nipbla antibody (*) at 24 hpf is not reproducibly observed and may be a degradation product of Nipbla. (B) Activities of *nipblb*-MOs at earlier stages. EGFP reporter RNAs (100 pg/embryo)—*nipbla*-5′-UTR-EGFP (upper panels) and *nipblb*-5′-UTR-EGFP (lower panels)—were injected alone or together with either *nipblb*-MO1 or *nipblb*-MO2 (1 ng/embryo), and EGFP fluorescence measured at 10–11 hpf. Views are lateral, with anterior and dorsal to the top and right, respectively. (C) Morphology of living embryos at indicated stages. Uninjected embryos, control embryos co-injected with 0.75 ng each of two 5-mis-*nipbl*-MOs, and embryos injected with either *nipbla*-MO1 (0.75 ng) or *nipblb*-MO1 (0.75 ng) alone or together (*nipbla/b*-morphants) are shown. Pericardial edema and tail defects are indicated by asterisks and arrowheads, respectively. Views are lateral, with dorsal to the top. Scale bar: 100 µm.

To analyze Nipbl function, we designed pairs of translation-blocking morpholino antisense oligonucleotides (MOs) for *nipbla* (*nipbla*-MO1 and MO2) and *nipblb* (*nipblb*-MO1 and MO2) that target regions in the 5′-UTR of each mRNA (see Materials and Methods). To evaluate MO efficiency and specificity, we made antibodies specific to Nipbla and Nipblb, and used them to quantify protein levels by Western blotting. Both proteins were detected at the predicted sizes, >300 kDa and 260 kDa, respectively (Figure S3A). Injection of 0.5 ng/embryo of *nipbla*-MO1 efficiently depleted Nipbla protein at 10 and 24 hpf, whereas *nipbla*-MO2 was much less efficient (Figures 2A, S3B,D). Injection of 0.5–1 ng of *nipblb*-MO1 partially depleted Nipblb protein at 10 hpf, but almost completely eliminated the protein by 24 hpf (Figures 2A, S3B,C). Higher amounts of *nipblb*-MO1 did not further deplete Nipblb protein at 10 hpf, nor did *nipblb*-MO2 (Figure S3B,D), even though both MOs were highly effective at 24 hpf (Figures 2A, S3C). As both MOs completely suppressed EGFP expression when co-injected with *nipblb*-5′-UTR-EGFP mRNA (Figure 2B), the data suggest that, at 10 hpf, a substantial fraction of Nipblb (but not Nipbla) is resistant to translational knockdown (e.g., there may be a relatively stable pool of maternal Nipblb protein).

Embryos co-injected with 0.75 ng each of 5-base mismatch control MOs (referred to as “control” embryos below) to either gene were indistinguishable from uninjected embryos (Figure 2C). Embryos injected with either *nipbla*-MO1 (0.75 ng) alone (*nipbla*-morphants) or together with *nipblb*-MO1 (0.75 ng; *nipbla/b*-morphants) resembled controls at 18.5 hpf, but began to exhibit defects by 24 hpf, including pericardial edema and a short tail (Figure 2C). At 34 hpf, *nipbla*-morphants had more severe pericardial edema (Figure 2C, asterisk) and no blood circulation (52.7%, *n* = 55) (Table S2). Some embryos had short tails, often split or branched along the dorsal-ventral axis (49.1%), which became more obvious at 52 hpf (Figure 2C, arrowheads). Embryos injected with *nipblb*-MO alone (0.75 ng; *nipblb*-morphants) appeared normal at 34 or 52 hpf, but co-injection of this MO with *nipbla*-MO1 increased the percentage with pericardial and tail defects (Figures 2C, S4B, Table S2). Some *nipbla/b*-morphants also had defects in their urogenital openings at 52 hpf (10.0%, *n* = 30) (Table S2, Figure S4D). In addition, Alcian staining at 120 hpf revealed changes in size, but not patterning, of most craniofacial cartilages, with particularly severe reductions of the hyosymplectic cartilage in the dorsal hyoid arch (pharyngeal arch 2; Figure S5).

**Figure 3:**
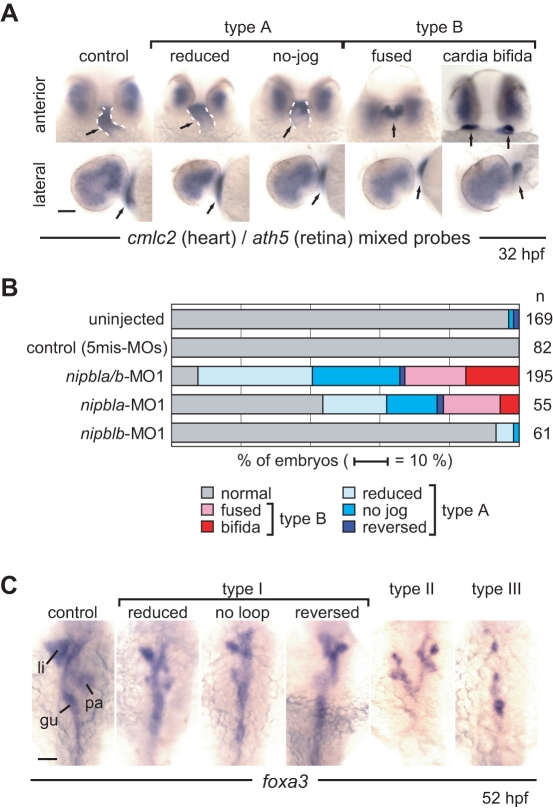
Effects of *nipbl*-MOs on heart and gut development. (A) Heart morphology was assessed at 32 hpf by *cmlc2* expression. Head-heart regions of control embryos (leftmost panels) and *nipbla/b*-morphants are shown in anterior (upper panels) and lateral (lower panels) views. Type A embryos form heart tubes (dashed white lines) but jogging to the left is incomplete (“reduced”) or absent (“no-jog”). Type B embryos fail to form heart tubes, and fusion of cardiac precursors at the midline is partial (“fused”) or entirely lacking (“cardia bifida”). Simultaneous detection of *ath5* expression throughout the dorsal and temporal retina in all embryos indicates no significant developmental delay in *nipbla/b-*morphants. Arrows point to heart tube or cardiac precursors. (B) Frequencies of different heart phenotypes in morphants; numbers (*n*) of embryos are on the right. (C) Gut and visceral organ morphology was assessed at 52 hpf by *foxa3* expression. Dorsal views, with anterior to the top, are shown for control embryos (leftmost panel) and *nipbla/b*-morphants. Type I embryos have thin gut tubes as well as small liver and pancreas. Looping of the gut tube is also reduced, absent (no loop), or reversed. In type II embryos, anterior gut tubes are split, and associated visceral organs are bilaterally duplicated. Type III embryos have few or no cells expressing *foxa3*, and lack a gut tube. Scale bars: 50 µm.

Many *nipbla/b*-morphants had heart defects, as visualized by ISH for a cardiac muscle marker, *cmlc2*, at 32 hpf (Figure 3A, Table S2). These were classified into two types: Type A, abnormal jogging/looping; and type B, defective cardiac precursor migration. Type A embryos (59% of *nipbla/b*-morphants) had beating hearts but showed reduced or no jogging to the left (Figure 3B, Table S2). Type B embryos (33% of *nipbla/b*-morphants) never formed a midline heart tube, and nearly half exhibited cardia bifida (Figure 3A–B, Table S2). This was not simply due to delayed development of morphants, as similar phenotypes were observed at 48 hpf (Table S2 and Figure S6A), and other developmental events, such as the spreading of *ath5* expression in the retina, occurred on schedule (Figure 3A). Similar heart defects were also observed in *nipbla* single morphants but at lower frequency (Table S2 and Figure 3B).

Because morphants with abnormal heart morphologies showed circulation defects (Figure S7A), we examined development of their blood/vascular system. O-dianisidine staining (which reveals differentiated erythrocytes; Figure S7B–E) and ISH for *gata1* (which labels erythrocyte precursors; Figure S7F,G) indicated that erythrocytes form normally, but accumulate in the ventral tail. Blood vessels, as marked by *fli1a*, are also specified normally in morphants (Figure S7H,I). These data suggest that circulation defects, and probably pericardial edema, in *nipbla/b*-morphants are due to impaired heart function.

*nipbla/b*-morphants also displayed defects in the looping of gut and visceral organs. ISH for the endodermal marker *foxa3* revealed a range of phenotypes, which we grouped into three classes (Types I–III; Figure 3C). At 52 hpf, the gut normally loops leftward, and liver and pancreas buds form on the left and right sides, respectively. Of *nipbla/b*-morphants, 64.5% (*n* = 96) had thin and abnormally looped guts, and a smaller liver and pancreas (Type I; Figure 3C). This included 38.5% with partial looping, 25% with no looping, and 1% with reversed looping. In type II embryos (11.5%), both the anterior gut and visceral organs were bifurcated or duplicated bilaterally, whereas in type III embryos (18.9%), the number of *foxa3*-expressing cells was severely reduced, and anterior gut tubes did not form (Figure 3C). Similar heart and visceral organ defects were also observed in embryos injected with an independent set of MOs (*nipbla*-MO2 and *nipblb*-MO2), although at lower frequency (Table S2).

### *nipbls* Regulate Endodermal Gene Expression

To gain insight into the earliest effects of *nipbl*-depletion on gene expression in the zebrafish embryo, we used microarrays to analyze mRNA from uninjected and *nipbla/b*-morphant embryos at early gastrula stages (6 hpf), hours before any morphological phenotypes become visible (Table S3). Partial loss of Nipbl function in mouse and man leads to many alterations in gene expression, most less than 1.5-fold [21],[23]. With effects in this range, large sample sizes (10–20 independent samples for each condition) are typically needed to achieve the statistical power to establish the significance of individual effects [21]. Due to sample limitations, our studies were restricted to three independent pools each of uninjected and morphant embryos. Therefore, we did not seek to infer significance directly from the data, but rather used them to generate ranked lists of candidate genes; up- or down-regulation of these was subsequently tested by quantitative-RT-PCR (Q-PCR). Thus, gene expression changes confirmed in the present study are most likely a subset of those that actually occurred.

**Figure 4:**
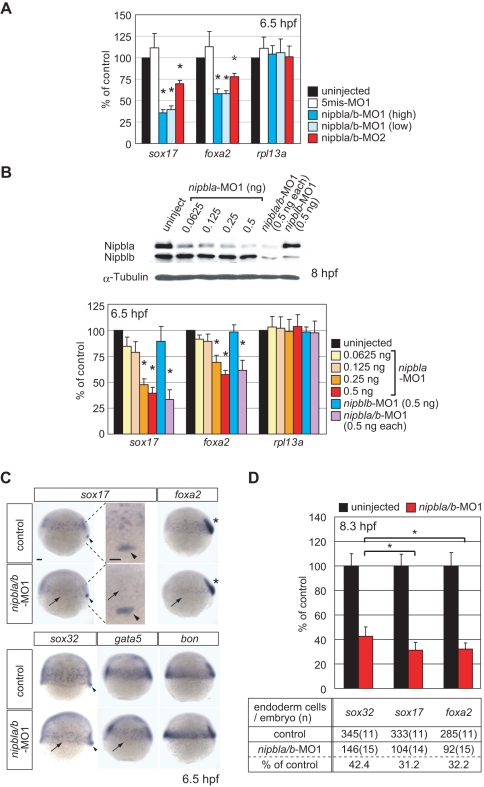
*nipbls* regulate the expression of endodermal genes. (A–B) Expression of *sox17*, *foxa2*, and *rpl13a* in *nipbla/b*-morphants at 6.5 hpf. mRNA levels were measured by Q-PCR, normalized to that of *ef-1a*, and expressed relative to values in uninjected embryos (*n* = 3, mean ± S.E.M.; * *p*<0.05 by paired *t* test). (A) Morpholino specificities. Uninjected embryos (black), control embryos injected with 2 ng each of 5mis-*nipbla*-MO1 and 5mis-*nipblb*-MO1 (white), and morphants co-injected with high doses (2 ng each; blue) or low doses (0.5 ng each; light blue) of *nipbla*-MO1 and *nipblb*-MO1, or with *nipbla*-MO2 (1.5 ng) and *nipblb*-MO2 (0.5 ng) (red). (B) Effects of single knockdown of Nipbla and Nipblb. Upper: protein levels of Nipbla and Nipblb at 8 hpf in morphants injected with indicated amounts of *nipbla*-MO1 and/or *nipblb*-MO1. Lower: Q-PCR analysis of *sox17*, *foxa2*, and *rpl13a* expression in uninjected embryos (black) and embryos injected with indicated amounts (0.0625–0.5 ng) of *nipbla*-MO1, with 0.5 ng of *nipblb*-MO1 alone, or with a mixture of 0.5 ng each of *nipbla*-MO1 and *nipblb*-MO1 (purple). (C) Analysis of endodermal gene expression by ISH. Control embryos (upper) and *nipbla/b*-morphants (lower) were analyzed at 6.5 hpf with *sox17*, *foxa2*, *sox32*, *gata5*, and *bon* Probes. Expression of all except *bon* was markedly (*sox17*, *foxa2*) or weakly (*sox32*, *gata5*) reduced in endoderm cells (arrows) of *nipbla/b*-morphants, but not in dorsal forerunner cells (*sox17* and *sox32*; arrowheads) or axial mesoderm (*foxa2*; asterisks), and this is more clearly shown in higher magnification panels of *sox17* expression at dorsal marginal regions. Views are lateral, with dorsal to the right, except the higher magnification panels (dorsal view, with anterior to the top.). Scale bar: 50 µm. (D) Endodermal cells, as detected by ISH with *sox32*, *sox17*, or *foxa2* probes, were counted in uninjected embryos (black) and *nipbla/b*-morphants (red) at 8.3 hpf (80% epiboly). The bar graph expresses the data relative to average numbers in uninjected embryos (mean ± SD; * *p*<0.001). The table underneath presents the average numbers of endodermal cells per embryo, along with the number of embryos counted (in parentheses).

As shown in Table S3, two known regulators of endoderm development, *sox17* and *foxa2*, appeared near the top of the list of potentially down-regulated genes. Q-PCR confirmed that both were significantly down-regulated by *nipbla/b-*MO1 even at minimum doses of these MOs (0.5 ng each), as well as by *nipbla/b*-MO2 (Figure 4A,B). The quantitative relationship between *nipbla* MO1 dose and *sox17/foxa2* reduction closely matched that between MO dose and Nipbla protein level (Figure 4B). Interestingly, the partial reduction in Nipblb protein caused by injection of *nipblb*-MO1 at this stage did not have a significant effect on the expression of these two endodermal genes, even in *nipbla*-morphants.

During gastrulation, *sox17* and *foxa2* are expressed not only in migrating endoderm, but also in dorsal forerunner cells (*sox17*) and axial mesoderm (*foxa2*). At 6.5 hpf (Figure 4C) and 8.5 hpf (Figure S8A), we consistently observed reduced expression of *sox17* and *foxa2* by ISH in the endoderm of *nipbla/b-*morphants (Figure 4C, arrows), but no significant change in their extra-endodermal expression (arrowheads). Thus, *nipbl* levels specifically affect endodermal expression of these genes.

*sox17* and *foxa2* are part of a pathway for endodermal specification that begins with Nodal signaling [34],[35]. They are induced by a zebrafish-specific *sox* gene, *sox32*, which is essential for the generation of endodermal cells [36]–[40]. We found that *sox32* expression was also decreased in the endoderm of *nipbla/b*-morphants (Figure 4C), although less severely than *sox17* or *foxa2*. Two genes are known to lie upstream of *sox32* in the endoderm specification pathway, *gata5* and *bon* [41]. By ISH, *gata5* expression was found to be slightly reduced at 6.5 hpf in *nipbla/b*-morphants (Figure 4C), and more severely reduced at later stages (Figure S8A); no change in *bon* expression was seen (Figure 4C). In contrast, further upstream genes such as the Nodal-relative *cyclops* (*cyc*) and the essential Nodal receptor co-factor *one-eyed-pinhead* (*oep*) as well as genes involved in mesodermal development (*no tail*, *ntl*; *even-skipped-1*, *eve1*; *and T-box gene 16*, *tbx16*) or expressed ubiquitously (*ribosomal protein L13a*, *rpl13a* and *POU domain class 5 transcription factor 1*, *pou5f1*) were all normally expressed in *nipbla/b*-morphants (Figure S8B,C). Nodal targets were also not among those conspicuously altered in expression in the microarray studies (Table S3).

We next sought to determine whether the decrease in expression of *sox17* and *foxa2* in *nipbla/b*-morphants is a direct effect of reduced Nipbl function, or is indirectly mediated by the reduction in *sox32* expression. Sox32 lies upstream of *sox17* and *foxa2* in two distinct ways. First, Sox32 is required for endoderm specification, so that reduced *sox32* expression might lower *sox17* and *foxa2* levels simply by depleting the cells that transcribe these genes. Second, Sox32 is a direct transcriptional activator of *sox17* and *foxa2*. To test whether the changes in expression of *sox17* and *foxa2* in *nipbla/b*-morphants could be accounted for by either of these explanations, two types of experiments were done.

First, we directly counted endodermal cells at late gastrula stages in control and *nipbla/b*-morphant embryos (Figure 4D). Using *sox32* ISH as a marker, we observed a 58% decrease in endodermal cells. However, using either *sox17* or *foxa2* as markers, the numbers of endodermal cells that could be visualized were significantly lower (68%–69%, *p*<0.001). These results suggest that endodermal depletion contributes to, but is only part of the explanation for, the overall reduction in *sox17* and *foxa2* expression.

**Figure 5:**
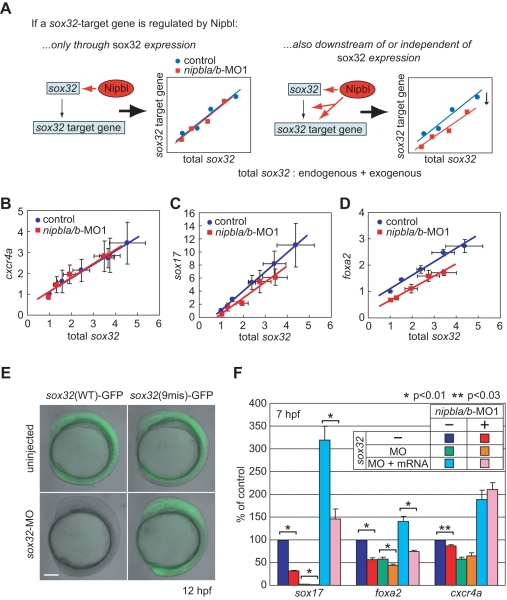
Multiple modes of regulation of *sox17* and *foxa2* by *nipbls*. (A–D) Embryos were injected with increasing (3.125–25 pg) amounts of in vitro synthesized *sox32* mRNA, and either 5-mis-*nipbl*-MO1 mixture (0.75 ng each: control) or *nipbl*-MO1 mixture (0.75 ng each: *nipbla/b*-MO1). mRNA levels for the *sox32*-target genes *sox17*, *foxa2*, and *cxcr4a* were quantified at 6.5 hpf by Q-PCR, normalized to *ef-1a*, and expressed relative to levels in embryos not treated with exogenous *sox32* mRNA. As *sox32* induces its own expression [38], target gene expression was determined as a function of total *sox32* (endogenous and exogenous, both measured directly), and not just the amount of *sox32* RNA injected. (A) Top: expected behavior of a *sox32* target gene that is not itself affected by *nipbls*. The same relationship between *sox32* level and target gene expression should be observed in control and *nipbla/b*-morphant embryos. Right: expected behavior of *sox32* target gene that is independently acted upon by *nipbls* (either directly, or because *nipbls* control the expression of other inputs to the gene). In this case, the relationship between *sox32* level and target gene expression will be shifted in *nipbla/b*-morphant embryos. (B) *cxcr4a* displays the expected behavior of a *sox32* target gene that is not itself affected by *nipbls*. (C,D) *sox17* and *foxa2* display the expected behavior of *sox32* target genes that are independently sensitive to *nipbl* function. In panels B–D, data are presented as mean ± S.E.M (*n* = 3). Dose-response relationships were well fit by straight lines (least-squares regression yielded values of r^2^>0.95 in all cases). (E, F) To eliminate any indirect effects of Nipbl reduction on gene expression through *sox32*, endogenous Sox32 protein was removed with a *sox32*-MO (5 ng) and replaced with *sox32*-9mis mRNA (10 pg), lacking the MO binding site for *sox32*-MO, and endodermal gene expression was examined by Q-PCR. (E) Embryos coinjected with *sox32*-MO and either a wild-type *sox32*(WT)- or mutated *sox32*(9mis)-GFP reporter construct show that a 9-base-mutation is sufficient to escape suppression of translation by *sox32*-MO. Lateral views at 12 hpf. Scale bar: 100 µm. (F) Effects of Nipbl reduction on expression of endodermal genes (*sox17*, *foxa2*, and *cxcr4a*) were examined in control (blue, red), Sox32-deficient (MO; green, orange), and Sox32-restored (MO+mRNA; light blue, pink) embryos at 7 hpf (*n* = 4, mean ± S.E.M.; * *p*<0.01 and ** *p*<0.03 by paired *t* test). The data show that induction of *sox17* and *foxa2* by exogenous Sox32 is markedly Nipbl-dependent, whereas induction of a different Sox32 target, *cxcr4a*, is not.

Second, we directly analyzed the transcriptional regulation of *sox17* and *foxa2* by Sox32 in controls and *nipbla/b-*morphants (Figure 5A–D). In one set of experiments, we measured the dose-dependence of induction of *sox17* and *foxa2* in response to injected, exogenous *sox32* mRNA. For any gene that Nipbls influence solely via the indirect effect of altering *sox32* levels, we would expect to see an identical dose-response relationship, with respect to total, measured Sox32 level, in both controls and morphants (Figure 5A, left panel). For *cxcr4a*, this is indeed what was observed (Figure 5B). In contrast, for *sox17* and *foxa2*, the dose-response curves were shifted downward in *nipbla/b*-morphants (Figure 5C,D). This implies a Nipbl-sensitive input to the expression of these genes, independent of the effect of the level of *sox32* (Figure 5A, right panel). In a second set of experiments, we effectively removed endogenous Sox32 with a *sox32*-MO, and replaced it with an exogenous *sox32* mRNA that lacked the MO-binding site (*sox32*-9mis) (Figure 5E,F). As expected, injection of the *sox32*-MO significantly reduced expression of *sox17*, *foxa2*, and *cxcr4a*, and this could be restored by co-injection of *sox32*-9mis mRNA. For *cxcr4a*, rescue of expression by *sox32*-9mis mRNA occurred to about the same degree in *nipbla/b*-morphants and control embryos. In contrast, for *sox17* and *foxa2*, exogenous *sox32* mRNA restored gene expression about half as well in *nipbla/b*-morphants as in controls (Figure 5F). These results imply that Nipbls influence the responsiveness of *sox17* and *foxa2* to transcriptional activation by *sox32*. Although it is possible that this effect reflects an influence of Nipbls on the expression of some transcriptional co-regulator of *sox17* and *foxa2*, the only other well-characterized activator of *sox17* in zebrafish is Pou5f1/Oct4 [42]–[44], and neither microarray analysis (Table S3) nor Q-PCR (Figure S8C) showed a change in *pou5f1* levels in *nipbla/b*-morphants. Thus, Nipbls may act directly upon *sox17* and *foxa2*.

### Functional Consequences of Altered Endodermal Gene Expression

Although the heart and gut derive from different germ layers (mesoderm and endoderm, respectively), Nipbls could regulate their development through common mechanisms. For example, mutations that affect early L/R patterning can cause defects in the looping of both heart and gut tubes [45]–[47], while mutations that affect early endoderm can severely disrupt medial migration of cardiac progenitors (which use the endoderm as a migratory substrate), leading in some cases to cardia bifida [36],[38],[48].

Interestingly, we observed that most morphants with type A heart jogging/looping defects later displayed type I gut looping defects (Figure S9A), consistent with both being caused by a common abnormality of L/R patterning. Similarly, most morphants with type B heart fusions, including those with cardia bifida, later displayed type III gut defects (Figure S9A), consistent with both types arising from a deficiency in early endoderm. This idea was further supported by the fact that type A and type B heart phenotypes were obtained at doses of *nipbla*-MO similar to those that caused type I and type II/III gut phenotypes, respectively (Figure S9B–D). Type B heart and type II/III gut phenotypes required at least 0.25 ng of *nipbla*-MO1 (Figure S9C,D), similar to the doses required for endodermal gene expression defects (Figure 4B). In contrast, looping defects were more sensitive to small changes in Nipbla protein levels (Figure S9C,D), as were circulation and tail defects (Figure S9E).

**Figure 6:**
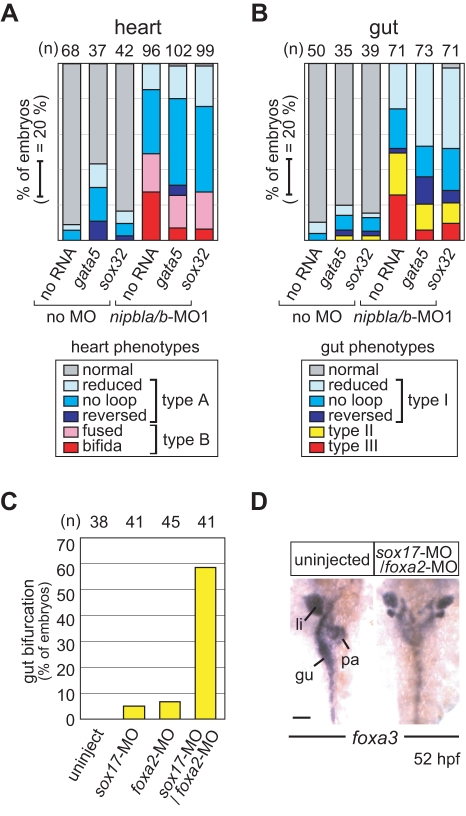
Nipbls affect gut and heart development through regulation of endodermal gene expression. (A, B) Exogenous *gata5* and *sox32* rescue a subset of gut and heart defects in *nipbla/b*-morphant embryos. mRNAs for *gata5* (10 pg) or *sox32* (2.5 pg) were injected into control embryos (no MO; left 3 columns) or *nipbla/b*-morphants (right 3 columns), and heart (A) and gut (B) morphologies were examined by ISH with *cmlc2* (32 hpf) and *foxa3* (52 hpf) probes, respectively. Numbers of embryos examined are shown at the top. (C, D) Gut bifurcation, similar to the type II gut phenotype found in *nipbla/b*-morphants, was induced by simultaneous reduction of *sox17* and *foxa2*. (C) Frequency of gut bifurcation in *sox17* and *foxa2*-single and double-morphants at 52 hpf. (D) Gut/visceral organ morphologies analyzed by ISH with *foxa3* probe. View is dorsal, with anterior to the top. Scale bar: 50 µm.

To test the hypothesis that endoderm deficiency is the cause of type B heart and type III gut phenotypes in *nipbla/b*-morphants, we attempted to rescue these defects by expressing exogenous *gata5* or *sox32* mRNA, which increases the number of endodermal cells in the gastrula-stage embryo [36],[37],[48]. At low levels of *gata5* or *sox32* mRNA expression, which did not themselves cause substantial heart or gut phenotypes, we observed marked rescue of both type B heart, and type III gut, phenotypes, but no significant rescue of type A (heart) or type I (gut) phenotypes (Figure 6A,B). These results support the idea that some heart and gut phenotypes in *nipbla/b*-morphant phenotypes have a common origin in reduced endodermal cell production or survival.

Interestingly, the type II gut phenotype (bifurcation with visceral organ duplication) was also partially rescued by exogenous *gata5* and *sox32* (Figure 6B). Gut/visceral organ duplication can arise from delayed medial migration of endoderm [49],[50]. Moreover, in mice, anterior gut duplications can be caused by loss of *Foxa2* [51],[52]. In zebrafish, however, gut defects have not been reported in *foxa2* mutants [53], but *foxa2* morphants do show subtle morphological changes in the liver and pancreas [54]. Upon repeating such studies, we observed a type II phenotype in a small proportion of *foxa2*-morphants (7%; Figure 6C). We also observed a type II phenotype in a small proportion (5%, Figure 6C) of *sox17*-morphants (*sox17* knockdown has not previously been reported, Figure S10). Intriguingly, when *sox17* and *foxa2* were knocked down simultaneously, the fraction of embryos displaying gut/visceral organ duplication rose to nearly 60% (Figure 6C). Yet even in such embryos, the sizes of liver and intestine were normal, or only slightly reduced (Figure 6D), in contrast to the marked reductions seen in most *nipbla/b*-morphants (Figure 3C). Thus, reduced expression of *sox17* and *foxa2* can synergistically reproduce most, but not all, aspects of the type II phenotype.

### Requirements for *nipbls* in Expression of Genes Involved in Left-Right Asymmetry

**Figure 7:**
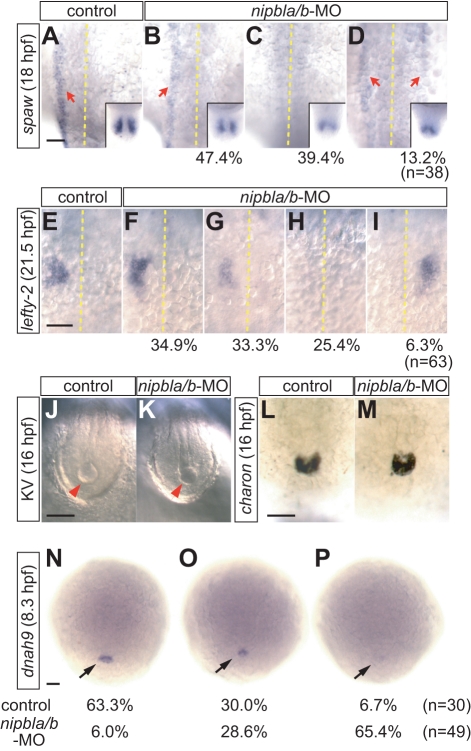
L/R patterning defects in *nipbla/b*-morphants. Left-sided expression of *spaw* (*n* = 38) and *lefty2* (*n* = 60) in lateral plate mesoderm (LPM) is disrupted in *nipbla/b*-morphants. (A–D) At 18 hpf (18-somite), *spaw* expression (red arrows) was variably unchanged (B; 47.4% of embryos), absent (C; 39.4%), or bilateral (D; 13.2%). The midline is marked by a dotted line. Insets show bilateral expression of *spaw* in the tail-bud. (E–I) *lefty2* expression at 21.5 hpf (25-somite) was either unchanged (F; 34.9%), reduced (G; 33.3%), absent (H; 25.4%) or right-sided (I; 6.3%). Views are dorsal, anterior to the top. (J–M) Morphology of Kupffer's vesicle (J, K; arrowheads; bright-field microscopy) and *charon* expression (L, M; ISH; posterior views of tail regions) are similar in control and *nipbla/b*-morphants at 16 hpf (14-somite). (N–P) Expression of *dnah9* in dorsal forerunner cells (arrows) at 8.3 hpf (80% epiboly) is reduced in *nipbla/b*-morphants (O, P) relative to controls. Views are dorsal, anterior to the top. Scale bars: 50 µm.

In contrast to other phenotypes, looping defects in heart (type A) and gut (type I) tubes were not significantly improved by expressing *gata5* and *sox32* (Figure 6A,B). Since, as mentioned above, these phenotypes could be caused by global defects in laterality, we examined expression of genes involved in L/R patterning [46],[47],[55],[56]. At 18 hpf, expression of the *nodal*-related gene *southpaw* (*spaw*) is normally restricted to the left lateral plate mesoderm (LPM) along the midline, as well as to a region adjacent to Kupffer's vesicle (KV), a structure involved in the initiation of L/R asymmetric patterning (Figure 7A) [57],[58]. In *nipbla/b*-morphants, *spaw* expression in LPM was severely reduced (Figure 7C) or present on both left and right sides (Figure 7D); in both cases, expression around KV was reduced (Figure 7C,D). A second nodal relative, *lefty-2*, is downstream of *spaw* [56] and is normally expressed in left LPM in the heart region at 21.5 hpf (Figure 7E). In *nipbla/b*-morphants, its expression was also severely reduced (Figure 7G) or lost (Figure 7H). Ectopic (right-sided) *lefty-2* expression was also observed in a small number of the morphants (Figure 7I).

The expression of *spaw* in left LPM is induced by signals from KV [58], and inhibited by the Cerberus-related protein Charon [59]. Although we did not observe morphological abnormalities in KV, or in *charon* expression (Figure 7J–M), expression of *dynein*, *axonemal*, *heavy polypeptide 9* (*dnah9*) was markedly reduced in most *nipbla/b*-morphants (Figure 7N–P). *dnah9* encodes a motor protein required for motility of monocilia in the KV, and its loss is known to impair KV fluid flow and disrupt L/R development [58]. This result suggests that Nipbls may act on L/R patterning by controlling KV function. On the other hand, the fact that the phenotypes of *nipbla/b*-morphants and *dnah9*-morphants are qualitatively different—*dnah9* has more of an effect on the sidedness, than the level, of *lefty-2* and *spaw* expression [58], whereas for *nipbls*, the opposite appears to be the case (Figure 7)—also raises the possibility that Nipbls exert later, more direct effects on the expression of left-side-specific genes. Consistent with this possibility, both *nipbla* and *nipblb* expression was detected in cells around KV and in LPM (both sides) at 14 hpf (10-som) and 20 hpf (22-som), respectively (unpublished data).

### Effects of Nipbl Deficiency Are Distinct from Those of Cohesin Deficiency

Nipbl/nipped-B/Scc2 was initially characterized as a cohesin-loading factor, and localizes extensively with cohesin on chromosomes (e.g., [60]). Whereas NIPBL mutations are responsible for more than half of CdLS cases, recent work has shown that CdLS can also be caused by mutations in the genes encoding cohesin subunits Smc1 and Smc3 [61],[62]. These and other findings suggest that Nipbl and cohesin work together in regulating gene expression [5],[18],[60],[63],[64]. Recently, it has been found that mutation and/or morpholino knockdown of cohesin function in the zebrafish produces a variety of gene expression changes, with a phenotype characterized by loss of expression of *runx3*, loss of hematopoietic expression of *runx1*, and concomitant lack of development of differentiated blood cells [32],[65].

**Figure 8:**
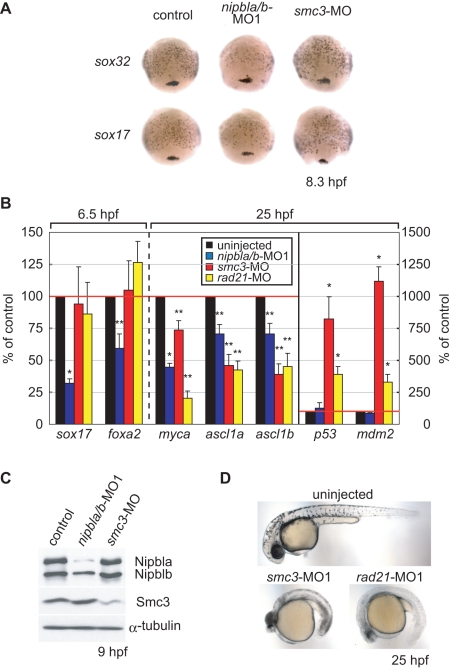
Distinct effects of Nipbl and cohesin on gene expression. (A) Expression of *sox32* (upper) and *sox17* (lower) in uninjected (left column), *nipbla/b*-morphants (middle column) and *smc3*-morphants (right column) was examined by in situ hybridization at 8.3 hpf (80% epiboly). Views are dorsal with anterior at top. (B) Gene expression changes in *nipbla/b*- and cohesin-morphants. Gene expression was examined in embryos injected with *nipbla/b*-MO1, *smc3*-MO, and *rad21*-MO by Q-PCR at 6.5 hpf or 26 hpf (as indicated). Expression levels are presented relative to those in uninjected embryos (100%; *n* = 3, * *p*<0.01, ** *p*<0.05). (C) Levels of Smc3, Nipbla, and Nibplb protein were examined by Western blotting at 9 hpf. α-Tubulin was used as a loading control. Amounts of MO injected in each embryo were: *nipbla/b*-MO1, 0.75 ng each; *smc3*-MO, 4 ng; *rad21*-MO, 5 ng. (D) Gross morphologies of cohesin-morphants at 25 hpf. Views are lateral with anterior to the left.

To address whether developmental effects caused by *nipbl* deficiency in the zebrafish could be explained by impaired cohesin function, we examined the effects of knocking down expression of cohesin subunit genes *smc3* and *rad21* (Figure 8) [31],[65]. Remarkably, expression of the endodermal genes *sox32*, *sox17*, and *foxa2*—which are markedly affected in *nipbla/b*-morphants—was unchanged in *smc3* or *rad21* morphants (Figure 8A,B), even at levels of MO that produced substantial reduction in cohesin protein level (Figure 8C) and caused gross morphological abnormalities (Figure 8D). Conversely, upregulation of *p53* and *mdm2*, which occurs in *smc3*- and *rad21*-morphants (Figure 8B; [65]), was not seen in *nipbla/b* morphants. For some genes, including *myca*, *ascl1a*, and *ascl1b*, expression was reduced in both types of morphants (Figure 8B).

Both *p53* and *mdm2* are known to be induced by a range of physiological stresses [66], suggesting that their differential induction in cohesin (*smc3* or *rad21*)- and *nipbla/b*-morphants might be due to different levels of overall phenotypic severity (and, thus, non-specific stress) in the two situations. To investigate this possibility, we injected lower amounts (0.75 ng/embryo) of *smc3*-MO (Figure S11A, low-*smc3*-morphants) and examined gene expression (Figure S11B). At levels at which reduction of both gross morphology and *ascl1a* and *ascl1b* expression were comparable between these and *nipbla/b*-morphants, induction of *p53* and *mdm2* was still much higher in the *smc3*-morphants, suggesting specific regulation by cohesin. These results, together with the observation that blood cells develop normally in *nipbla/b* morphants (Figure S7), suggest that *nipbl* and cohesin have overlapping, but distinct, influences on gene expression.

## Discussion

### Zebrafish as a Model Organism for the Study of CdLS

**Figure 9:**
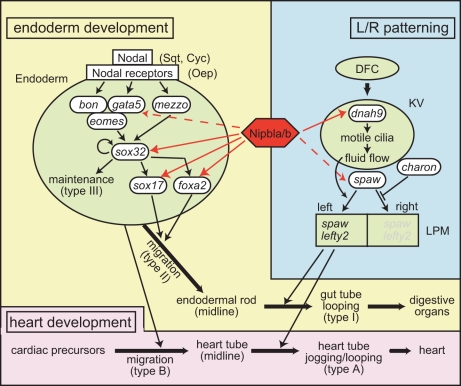
Multiple influences of Nipbls on regulatory networks underlying heart and visceral organ development. Endoderm development is initiated by Nodal-dependent activation of a network of transcription factors. Within this network, Nipbls regulate expression of *gata5*, *sox32*, *sox17*, and *foxa2*. Sox32 is required for specification and maintenance of endoderm cell fate, and reduced levels of Sox32 in *nipbla/b*-morphants may reduce endodermal cell number. This, in turn, could produce in a thinner gut, and smaller visceral organs, as well as a failure of cardiac progenitors that migrate along endoderm to form a midline heart tube. In parallel, a network triggered by cilia-driven fluid flow in KV underlies the establishment of L/R asymmetry in the embryo. Within this network, Nipbls regulate expression of *dnah9*, as well as the asymmetric expression of *spaw* and *lefty2*. Disruption in the expression of these genes is likely to underlie the heart jogging/looping, and gut looping defects in *nipbla/b*-morphants.

Here we characterize the zebrafish *nipbl* genes, and show that *nipbl*-morphant zebrafish display multiple abnormalities in early heart and gut/visceral organ development (Figure 3). These changes are preceded by reduced expression of genes required for endoderm formation and function, and L/R patterning, both processes being critical for normal heart and gut development (Figures 4,7; Table S2). We demonstrate that at least some of the observed gene expression changes are sufficient, collectively, to produce a subset of the observed morphological abnormalities (Figure 6C,D). These results indicate that heart and gut/visceral organ deficits arise in the context of both abnormal endoderm development and abnormal L/R patterning (Figure 9).

The parallels between *nipbl*-morphant phenotypes and the birth defects in CdLS [16] are striking. Heart and gut abnormalities are prominent in CdLS, and among the primary causes of morbidity and mortality [16],[17],[67]. Marked L/R asymmetry in the severity of defects in CdLS (e.g., in limbs; [17]), together with the occurrence of intestinal malrotation [16], suggest that overall L/R patterning is also disturbed in this syndrome. Gut duplications, which are sometimes observed in *nipbl*-morphant fish (Figure 3, Table S2), are also seen, rarely but significantly, in CdLS [68],[69]. As in CdLS, and in *Nipbl*-heterozygous mice [21], Nipbl-deficient fish also exhibit growth retardation and distinctive craniofacial abnormalities (Figures 2C and S5), the latter including severe reductions of the hyosymplectic cartilage, the homologue of the mammalian stapes [70]. Abnormal development of the stapes and other middle ear bones is reported in CdLS [71], and our results suggest that defects in embryonic development of the precursors of these bones could account for some aspects of hearing loss in CdLS [16],[17] and in *Nipbl*-heterozygous mice [21].

Notwithstanding some major anatomical differences between fish and mammals (e.g., fish hearts are not divided by septa), these results suggest that developmental alterations caused by Nipbl deficiency in zebrafish may provide mechanistic insight into the origins of human birth defects. For example, the results immediately raise the possibility that heart and gut defects in CdLS originate during gastrulation, much earlier than might have been suspected from the times at which structural abnormalities (e.g., septal defects) are observed [21]. One reason why *nipbl*-morphant zebrafish may provide such a good model for CdLS is that the early insensitivity of *nipblb* to MO-mediated knockdown (Figures 2, S3) may fortuitously make the total decrease in Nipbl function in *nipbla/b*-morphant embryos approximate what occurs in human and murine *NIPBL*-haploinsufficiency [21],[23]. This view is supported by data suggesting that Nipbla and Nipblb have similar functions, e.g. knockdown of *nipblb* in *nipbla*-single morphants increases the frequency of heart defects. Yet the fact that Nipblb reduction does not also increase the frequency of endodermal gene expression defects suggests that some functional differences exist between the two genes.

Despite the fact that, at late stages, both Nipbla and Nipblb proteins were strongly reduced in *nipbla/b*-morphants, their phenotypes were much milder than those of *smc3* or *rad21*-morphants, suggesting that only trace amounts of Nipbls are sufficient to maintain normal chromosomal cohesion. Consistent with this view, *nipbla/b*-morphants display at most a small increase in premature sister chromatid separation (unpublished observations), similar to what is seen in *NIPBL*-heterozygous human and mouse cells [21],[23],[25].

### Regulation of Gene Expression by *nipbls*

It is rapidly emerging that Nipbl and cohesin play global, if still poorly understood, roles in the regulation of gene expression (e.g., [1],[5],[21],[23],[27],[72]–[74]). In human, mouse, and fly cells, partial reduction in *NIPBL/Nipped-B* function produces modest changes in the expression of large numbers of genes [21],[23],[27]. In *nipbla/b*-morphant zebrafish, we also observed modest changes in the expression of multiple zygotic genes (Figures 4,7,S8; Table S3). Because these changes were detected at early gastrula stages—only hours after the onset of zygotic transcription—the probability that some of them are directly caused by reduced Nipbl function should, *a priori*, be greater than in studies using other organisms, in which many gene expression changes could be secondary effects of other gene expression changes. Interestingly, a subset of the genes identified by microarray experiments as potentially affected by Nipbl depletion in zebrafish (Table S3) also show changes in Nipbl heterozygous mouse fibroblasts (*pitx2*, *cebpd*, *jag1* [21]), embryonic mouse brain (*alcam*, *cnot8*, *cxcl12*, *hlcs*, *myc*, *neo1*, *nos1*, *notch2*, *vldlr* [21]) or human lymphoblastoid cells (*alcam*, *myc*, *bmi1*, *ctage5*, *id3*, *nsun2*, *pccb*, *psme1*, *ptma*, *rab2a*, *robo1*, *rora*, *sfrs1*, *snrp70*, *snx3*, *ube2g1* [23]). Whether such overlap is functionally significant is difficult to assess given the different tissues and stages examined in different organisms.

Interestingly, we found some of the genes dysregulated in *nipbl*-morphants to be members of known gene-regulatory networks: e.g., *gata5*, *sox32*, *sox17*, and *foxa2*, controlling endoderm development; and *dnah9*, *spaw*, and *lefty2*, controlling L/R patterning. By examining the effects of graded *sox32* misexpression as well as replacement of endogenous with exogenous Sox32, we showed that changes in *sox17* and *foxa2* expression due to Nipbl deficiency are only partially explained by altered levels of *sox32*, their common upstream activator (Figures 4–5), and presented evidence that Nipbls influence the transcriptional responsiveness of *sox17* and *foxa2*. Consistent with this, preliminary chromatin immunoprecipitation studies suggest that Nipbla binds near the transcriptional start site of *sox17* (unpublished observations). Whether Nipbls regulate responsiveness of *sox17* and *foxa2* solely to Sox32, or to other transcriptional effectors as well, is not known, but specificity is suggested by the fact that extra-endodermal patterns of expression of *sox17* and *foxa2* are largely insensitive to Nipbl depletion (Figures 4C, 7A). *foxa2* expression in floor plate, for example, is regulated by Nodal and Shh, but independent of Sox32 [53], and is normal in *nipbla/b*-morphants (Figure S8 and unpublished observations).

The mechanisms by which Nipbl regulates gene expression are currently unknown. *Drosophila* Nipped-B and cohesin occupy largely overlapping sites in the genome [60]. In mammalian cells, most cohesin localizes to binding sites for the insulator protein CTCF, which requires cohesin for function [75]–[78], but a recent study of mammalian cells [5] indicates that Nipbl preferentially occupies non-CTCF sites at promoter regions, at which complexes exist between cohesin and the Mediator complex (which mediates transcriptional transactivation). Our finding that *nipbl*-knockdown and *cohesin*-knockdown produce distinctive phenotypic and gene-expression effects in the zebrafish (Figure 8) strongly suggests that only some of the functions of Nipbl are attributable to a general role in promoting cohesin function. In *Drosophila*, manipulation of Nipped-B and cohesin expression has also been observed to have overlapping, different, or even opposite effects, depending upon the experimental circumstances [24],[27],[64]. In man, the mild forms of CdLS that have been linked to mutations in cohesin subunit genes (Smc1 and Smc3 [61],[62]) appear to be caused by specific, rare missense mutations (i.e., very likely not simple loss-of-function alleles), also consistent with the idea that the cause of CdLS is not simply a reduced level of cohesin function, but rather the selective disruption of specific functions in which Nipbl and cohesin work together. Such functions could be related to the ability of Nipbl to interact directly with molecules such as histone deacetylases, HP-1γ, and chromatin remodeling factors (e.g., [29],[30],[79]). Indeed, given its large size and conserved, multi-domain structure, it may turn out to be more appropriate to envision Nipbl as a cohesin-associated scaffold protein, rather than a cohesin-loading factor.

### From Gene Expression to Developmental Phenotypes

Endoderm development and L/R patterning are crucial events in normal heart and gut development (Figure 9), and both are clearly affected in *nipbla/b*-morphants. Endoderm development appears to be impaired at more than one level, consistent with altered expression of multiple genes (*sox32*, *sox17*, *foxa2*, *gata5*) in the pathway controlling early endoderm specification and formation of the gut. Previous work suggests that *gata5* and *sox32* are required for specification of endodermal cell fates, whereas *sox17* and *foxa2* act downstream at the level of endodermal differentiation [37]–[39],[41],[48]. We hypothesize that reduced *gata5* and/or *sox32* expression is the cause of type III gut phenotypes (reduced gut/visceral organ tissue) in *nipbla/b*-morphants; consistent with this, injection of exogenous *gata5* or *sox32* mRNA rescues this phenotype (Figure 6B). A defective urogenital opening—which is also seen in *nibpla/b*-morphants (Figure S4)—is also a known consequence of a deficiency of endoderm [36],[80].

Exogenous *gata5* and *sox32* mRNA also rescued type B heart phenotypes (failure to form a normal midline heart tube; Figures 6A, S6), consistent with the known dependence of cardiac progenitor migration on the endoderm. Moreover, most aspects of the type II gut phenotype (anterior bifurcation/duplication) were efficiently produced by simultaneous MO-knockdown of *sox17* and *foxa2* (Figure 6C,D). These results are consistent with *sox17* and *foxa2* acting downstream of endoderm specification to influence endodermal cell behaviors, such as migration (Figure 9). In contrast, defects in *nipbla/b*-morphants characterized by abnormal or reversed looping (type I gut and type A heart defects) were not significantly rescued by *gata5* or *sox32* mis-expression, and only weakly reproduced by combined *sox17*/*foxa2* knockdown (Figure 6 and unpublished data). Given the nature of these phenotypes, we suspect they are primarily caused by a common defect in L/R patterning (Figure 7).

Interestingly, these different phenotypes correlate with different degrees of depletion of Nipbla protein levels; i.e., endoderm-related (type B heart and type II/III gut) phenotypes require more significant reduction of Nipbla protein than L/R patterning-related (type A heart and type I gut) phenotypes (Figure S9). However, since we examined protein levels in whole embryos, we cannot exclude the possibility that effects of MO on protein levels differ between cell types, and a higher amount of *nipbla*-MO may be required for significant reduction of Nipbla protein levels in endodermal cells.

Taken together, our findings imply a mechanistic model in which reduced Nipbl function has modest, quantitative effects on the expression of multiple genes; some of these changes lead to quantitative functional deficits; and these in turn contribute collectively to the appearance of developmental defects (Figure 9). For at least one set of genes (*sox17* and *foxa2*), the mode of gene interaction is synergistic; i.e., quantitative alterations in the levels of both genes seem to be necessary to produce at least one aspect of the *nipbl*-morphant phenotype (Figure 6C). If phenotypes in *nipbl*-morphant zebrafish are indeed explained by the cooperative effects of quantitative changes in gene expression, it not only provides a framework for understanding specific classes of birth defects syndromes, such as CdLS and the cohesinopathies; it also suggests a mechanism by which non-syndromic birth defects (which are far more common) might naturally arise out of combinations of quantitative genetic variants in the human population.

## Materials and Methods

### Ethics Statement

All animals were handled in strict accordance with good animal practice as defined by the relevant national and/or local animal welfare bodies, and all animal work was approved by the University of California, Irvine Institutional Animal Care and Use Committee.

### Fish Maintenance and Embryo Raising

Zebrafish (AB strain) were maintained and staged as described [81],[82].

### Cloning of Full-Length cDNA of nipbla and nipblb

By searching Genbank and Sanger DNA/protein databases using the human NIPBL protein sequence, we found entries for *nipbla* and *nipblb* genes. Although the Ensembl database had only partial sequences for both genes (ENSDART00000086861 and ENSDART00000086653 [*nipbla*]; ENSDART00000111663 and ENSDART00000108661 [*nipblb*]), these differed from those in the NCBI database (XM_001919812 [*nipbla*]; XM_001920168 [*nipblb*]) even within overlapping regions. We therefore cloned and sequenced cDNAs for both genes by RT-PCR, using cDNA from wild-type zebrafish embryos (9 hpf). cDNA fragments containing 5′-UTR were amplified by 5′ RACE, subcloned into the pCRII-TOPO vector (Invitrogen), and sequenced.

### Microinjection of Morpholino Antisense Oligo and mRNA

Morpholino antisense oligos (MO) were designed to block translation or splicing (Gene Tools, Inc.) including: *nipbla*-MO1, 5′-ACGTGGACGCACAGGTTGCTCAGTG-3′; *nipbla*-MO2, 5′-TCGCTGCTCACTGATCCACCTTTAC-3′; *nipblb*-MO1, 5′-TGACGGCTGGGCACAGAAGTCTAAC-3′; *nipblb*-MO2, 5′-GCACACAGAGATCCACAGAGATATT-3′; 5mis-*nipbla*-MO1, 5′-ACcTcGACGgACAGcTTcCTCAGTG-3′; 5mis-*nipblb*-MO1, 5′-TGACGcCTcGGCAgAGAAcTgTAAC-3′; *sox17*-MO, 5′-CCATGACTTACCTATAAACAGAACA-3′; *foxa2*-MO, 5′-CCTCCATTTTGACAGCACCGAGCAT-5′ [53]; *sox32*-MO, 5′-CAGGGAGCATCCGGTCGAGATACAT-5′ [38], *smc3-*MO, 5′-GTACATGGCGGTTTATGCACAAAAC-3′ [31]; *rad21*-MO, 5′- AGGACGAAGTGGGCGTAAAACATTG-3′ [32].

MOs were prepared at 20 mg/ml and diluted in 1× Danieau buffer (58 mM NaCl, 0.7 mM KCl, 0.4 mM MgSO_4_, 0.6 mM Ca (NCO_3_)_2_, 5 mM HEPES (pH 7.6)) and stored at −20°C. To construct *nipbla*-5′-UTR-EGFP and *nipblb*-5′-UTR-EGFP reporter genes, double stranded oligo DNAs encoding 5′-UTR of *nipbla* or *nipblb* containing target sites of MOs were fused with EGFP cDNA by subcloning both into the pCS2+ vector. Full-length cDNAs of *gata5* and *sox32* were amplified by RT-PCR and cloned into pCS2+. *sox32*-9mis cDNA was prepared by RT-PCR using the cloned *sox32* cDNA as a template with primers designed to introduce mutations within the *sox32*-MO target sequence located at 5′ end of ORF. Mutations in *sox32*-9mis are introduced within the first 25 bases of ORF: ATGTActTgGAtaGaATGtTgCCaG (mutations are shown in lower cases and do not change amino acid sequence). Effective translation of *sox32*-9mis in the presence of *sox32*-MO was examined by reporter assay using fusion reporter constructs, in which the first 30 bases of *sox32* and *sox32*-9mis ORF was fused in frame with EGFP cDNA (Figure 5E). Capped mRNA was synthesized using mMESSAGE mMACHINE kit (Ambion). MOs and in vitro synthesized mRNA were injected into yolk of embryos at the 1–4-cell stage.

### Whole Mount In Situ Hybridization (ISH) and Northern Blotting

Whole mount ISH was performed using digoxigenin (DIG)-labeled antisense RNA probes [83]. For Northern blotting, total RNA was prepared using TRIzol (Invitrogen). RNA (10 µg per lane) was separated on formaldehyde gels, transferred to nylon membranes, and probed with two different DIG-labeled antisense RNA probes for *nipbla* and *nipblb*. Hybridized probes were detected by using alkaline phosphate-conjugated anti-DIG antibody and visualized with NBT/BCIP (Roche).

### Western Blotting

Plasmids for expression of 6x-His-tagged fusions of amino acid residues 523–948 of Nipbla and residues 232–593 of Nipblb (corresponding to regions poorly conserved between the two proteins) were constructed by subcloning partial cDNA fragments of *nipbla* and *nipblb* into the pET15b vector. The proteins were expressed in *E. coli* (Rosetta-gami2 [Novagen]), purified by nickel-column chromatography, and used to immunize rabbits at Open Biosystems, Inc. For Western blotting, total protein was extracted from embryos as described [84]. Briefly, chorions were removed, yolks punctured by pipetting in 1/2× Ginzburg Fish Ringer without calcium (55 mM NaCl, 1.8 mM KCl, 1.25 mM NaHCO_3_), and cells collected by centrifugation and lysed by boiling in Laemmli buffer. Total protein (5–10 embryos per lane) was separated by SDS-PAGE and subjected to Western blotting with anti-Nipbla (1∶1,000), anti-Nipblb (1∶400), and anti-α-Tubulin (Sigma, 1∶2,000), and detected by chemiluminscence (SuperSignal, Pierce). Protein levels were quantified using ImageJ software.

### Quantitative RT-PCR (Q-PCR)

Gene expression was measured by Q-PCR using SYBR green and analyzed using iQ5 and CFX software (BioRad). cDNA was synthesized using Superscript III first strand synthesis (Invitrogen). All data were normalized to *ef-1a* as a reference, and *rpl13a* [85] served as a negative control for MO specificity (neither *ef-1a* nor *rpl13a* transcript levels were altered in microarray analysis of *nipbla/b*-morphants; unpublished data). At least three replicates were examined for each primer set and data were averaged over three independent experiments ± S.E.M. *p* values were calculated from *ΔCt* values by paired *t* test. Primers for Q-PCR are shown in Table S1.

### Microarray

RNA was prepared from 30–40 each of uninjected embryos or embryos co-injected with 2 ng each of *nipbla*-MO1 and *nipblb*-MO1. Total RNA was isolated at 6 hpf (Shield stage) using TRIzol and further purified using the RNeasy kit (Qiagen). cRNA preparation, hybridization, and scanning were done at the microarray facility at the University of California, Irvine using Affymetrix GeneChip Zebrafish Genome Arrays. Hybridization was performed in triplicate using cRNA from three biologically independent samples. Data were analyzed with Gene Pattern Web software (http://www.broadinstitute.org/cancer/software/genepattern/), and ranked by permutation analysis.

### Alcian Blue Staining

Embryos were fixed at 120 hpf and craniofacial cartilages were visualized by staining with Alcian blue as described previously [86].

## Supporting Information

Figure S1 Predicted amino acid sequences of zebrafish Nipbla and Nipblb. Predicted amino acid sequences of zebrafish Nipbla and Nipblb were aligned with human NIPBL (NP_597677). Conserved identical and similar amino acids are shown in dark and light purple, respectively. Predicted motifs, as shown in Figure 1A, are in the colored boxes: Regions required for binding to Scc4 (green), HP1 (red), and HDAC1 and 3 (blue), putative nuclear localization signal (yellow), and HEAT domains (black).
(TIF)

Figure S2 Expression of *nipbla* and *nipblb* mRNAs during development. Northern blot. Total RNA was prepared from embryos at indicated stages and loaded at 10 mg per lane. mRNAs were analyzed with DIG labeled antisense RNA probes (AS1 in Figure 1A). *ef-1a* was used as a control. Expression level of *ef-1a* was significantly increased after mid-blastula transition (3 hpf) as reported [87]. Molecular weights are indicated on the right.
(EPS)

Figure S3 Activities of *nipbl*-MOs. To evaluate MO specificity, we used two different MOs (MO1 and MO2) for each *nipbl* gene that target non-over-lapping sequences within the 5′-UTR, and used Western blotting with anti-Nipbla and Nibplb antibodies to find the minimum amount required for maximal knockdown. (A) Nipbla- and Nipblb-specific antibodies were prepared and evaluated by Western blotting using lysates of embryos at 10 hpf (10 embryos per lane). Molecular weight markers are on the right. (B) Dose-dependence of MO effects. *nipbla*-MO1 virtually eliminated Nipbla protein at 0.5 ng/embryo (upper left). *nipbla*-MO2 only partially suppressed Nipbla expression, even at 2–4 ng/embryo (lower left). Nipblb protein was reduced by either of two *nipblb*-MOs at 0.25–0.5 ng/embryo partially, but further reduction was not observed with higher amounts of MOs (2 ng/embryo) (upper and lower right). (C) Effects of *nipblb*-MOs on Nipblb protein levels at 24 hpf. Nipblb protein was significantly reduced by 0.5–1 ng/embryo of *nipblb*-MO1 and MO2 at 24 hpf. (D) Both Nipbla and Nipblb proteins were reduced in embryos co-injected with 2 ng each of *nipbla*-MO1 and *nipblb*-MO1 (*nipbl*-MOs), but not in uninjected embryos or embryos co-injected with 2 ng each of 5mis-*nipbla*-MO1 and 5mis-*nipblb*-MO1 (5mis-MOs). α-Tubulin is a loading control. Although 5mis-MOs at 2 ng each/embryo had no effect on Nipbl levels, they did cause delay of development after mid-somitogenesis (unpublished data). Accordingly, given these results, the minimum effective amounts of MOs were determined to be 0.5 ng/embryo (for *nipbla*-MO1, *nipblb*-MO1, and *nipblb*-MO2) and 1 ng (for *nipbla*-MO2).
(EPS)

Figure S4 Morphological abnormalities in *nipbla/b*-morphants. Lateral views of tails (A, B) and anal regions (C, D) of controls and *nipbla/b*-morphants at 52 hpf. Morphant tails are shortened and branched along the dorsal-ventral axis (B). Some morphants (10%) lack a urogenital opening (arrows in C and D). Scale bars: 50 µm.
(EPS)

Figure S5 Defects in the larval craniofacial skeleton in *nipbla/b*-morphants. Craniofacial cartilages were stained with Alcian blue at 120 hpf. Controls (A, D, G) and two examples of *nipbla/b*-morphants (B, C, E, F, H, I) are shown in ventral (A–C), ventrolateral (D), and lateral view (E–I), anterior to the left. Specific reductions in hyosymplectic cartilages are indicated by arrows (D–F) and outlined at higher magnification (G–I). Scale bar: 100 µm.
(TIF)

Figure S6 Effects of *nipbls* on heart development. Heart morphology in *nipbla/b*-morphants at 48 hpf was examined by the expression of *cmlc2*. Anterior views of control embryos (leftmost panel) as well as *nipbla/b*-morphants with type A or type B heart defects. White dotted lines outline the heart tubes and arrows indicate split hearts in type B morphants. Scale bar: 50 µm.
(EPS)

Figure S7 Circulation defects and pericardial edema in the *nipbla/b*-morphants. (A) Numbers of embryos with heart defects in *nipbla/b*-MOs within each class that showed circulation defects or pericardial edema at 32 hpf. (B–E) o-Dianisidine staining of developed erythrocytes. Erythrocytes were stained by incubating anesthetized living embryos in 10 mM sodium acetate, pH 4.5, 0.65% hydrogen peroxide, 40% ethanol, and 0.6 mg/ml of o-dianisidine for 15 min at 28.5°C. Control embryos (B, C) and *nipbla/b*-morphants with circulation defects (D, E) were stained with o-dianisidine at 32 hpf. Ventral views of the heart (B, D) and lateral views of the tail (C, E) are shown. In morphants, erythrocytes accumulated ventrally (arrow head in E) including the intermediate cell mass (ICM) (arrow in E), and yolk surface (D). (F–I) *gata1* (blood cells) (F, G; 20 hpf) and *fli1a* (blood vessels) (H, I; 31 hpf) expression were examined by ISH in controls (F, H) and *nipbla/b*-morphants (G, I). Scale bars: 100 µm.
(EPS)

Figure S8 Effects of reduction of *nipbls* on endodermal and Nodal-signaling genes. (A) Expression of endodermal genes (*sox17*, *foxa2*, and *gata5*) was examined by ISH at 8.5 hpf. Expression of all three was downregulated in endoderm (arrows) but not in dorsal forerunner cells (*sox17*; arrowheads) or axial mesoderm (*foxa2*; brackets). *gata5* expression was mainly reduced dorsally (arrow). (B) In contrast, no changes were observed in expression of the Nodal-related gene, *cyc*, a Nodal receptor co-factor, *oep*, or a mesodermal Nodal target, *ntl*, in *nipbla/b*-morphants compared with controls at 6.5 hpf. Lateral views with dorsal to the right in all cases except *gata5*, which is shown in dorsal view, anterior to the left. Scale bars: 50 µm. (C) Expression of endodermal and nonendodermal genes were examined by Q-PCR at 7 hpf in controls (black) and *nipbla/b*-morphants (red). Expression levels are normalized to that of *ef-1a* and shown relative to values in control embryos. *p* values were calculated by paired *t* test, using ΔCt values (*n* = 4, mean ± S.E.M., * *p*<0.002 and others are *p*>0.1).
(EPS)

Figure S9 Correlation between phenotypic variation and Nipbla protein level. (A) Frequencies of gut/visceral organ morphologies in *nipbla/b*-morphants, as examined by *foxa3* expression at 52 hpf, after live-sorting embryos at 32 hpf according to heart morphology. (B–E) A series of diluted *nipbla*-MO1 (0.031–0.75 ng as indicated) was coinjected with a fixed amount (0.75 ng) of *nipblb*-MO1, and protein levels (B) as well as phenotypes (C–E) were examined. (B) Protein levels were examined by Western blotting at 6.5 hpf (upper panels). Nipbla and Nipblb bands were quantified using ImageJ, normalized to α-Tubulin, and plotted relative to values in uninjected controls (*n* = 4, mean ± S.E.M). (C–E) Frequencies of heart defects at 32 hpf (C), gut defects at 52 hpf (D), and circulation and tail defects at 32 hpf (E).
(EPS)

Figure S10 Activity of *sox17*-MO. (A) Schematics of *sox17* pre- and mature mRNAs showing positions of the *sox17*-MO binding site and primers used for RT-PCR. (B) Effectiveness of a splice-blocking *sox17*-MO was examined by RT-PCR. cDNA from uninjected embryos and embryos injected with 2.5 and 5 ng of *sox17*-MO was prepared at 7 hpf and expression of pre- (a, c) and mature- (b) *sox17* mRNAs was examined by RT-PCR.
(EPS)

Figure S11 Effects of low amounts of *smc3*-MO on gene expression. (A) Morphology of living embryos at 26 hpf. Lateral views with anterior to the left. (B) Q-PCR analyses of gene expression in uninjected control (black), *nipbla/b*-morphants (blue), and low-*smc3* (0.75 ng)-morphants (red) at 25 hpf by Q-PCR (*n* = 3, mean ± S.E.M.; * *p*<0.05 and # *p*>0.1 by paired *t* test). Scale bar: 100 µm.
(EPS)

Table S1 Primers used for Q-PCR.
(DOC)

Table S2 Phenotypes of *nipbl*-morphants.
(DOC)

Table S3 600 genes potentially down- or up-regulated in *nipbla/b*-morphants.
(DOC)

## Abbreviations

CdLS: Cornelia de Lange Syndrome
DIG: digoxigenin
EGFP: enhanced green fluorescent protein
ISH: in situ hybridization
KV: Kupffer's vesicle
L/R: left-right
MO: morpholino
Nipbl: Nipped-B-like
NLS: nuclear localization signal
Q-PCR: quantitative RT-PCR
RT-PCR: reverse transcription polymerase chain reaction
UTR: untranslated region
