# Supplementary figures and images for: Multifactorial Origins of Heart and Gut Defects in nipbl-Deficient Zebrafish, a Model of Cornelia de Lange Syndrome
Source: PLoS Biol. 2011 Oct 25;9(10):e1001181. doi: 10.1371/journal.pbio.1001181 (PMC3201921; doi:10.1371/journal.pbio.1001181)

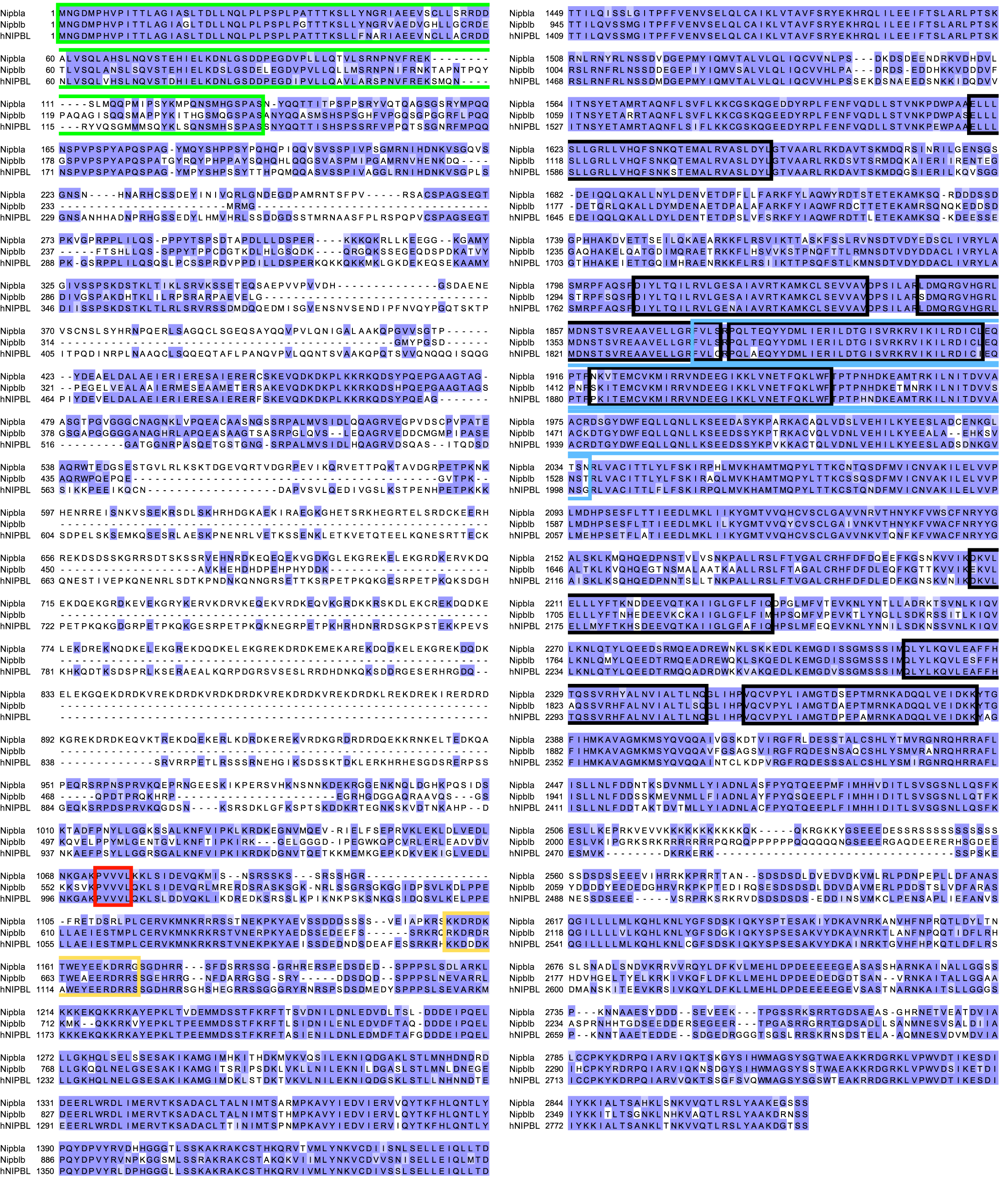

Supplement: Figure S1 — Predicted amino acid sequences of zebrafish Nipbla and Nipblb. Predicted amino acid sequences of zebrafish Nipbla and Nipblb were aligned with human NIPBL (NP_597677). Conserved identical and similar amino acids are shown in dark and light purple, respectively. Predicted motifs, as shown in Figure 1A, are in the colored boxes: Regions required for binding to Scc4 (green), HP1 (red), and HDAC1 and 3 (blue), putative nuclear localization signal (yellow), and HEAT domains (black). (TIF) [file pbio.1001181.s001.tif]

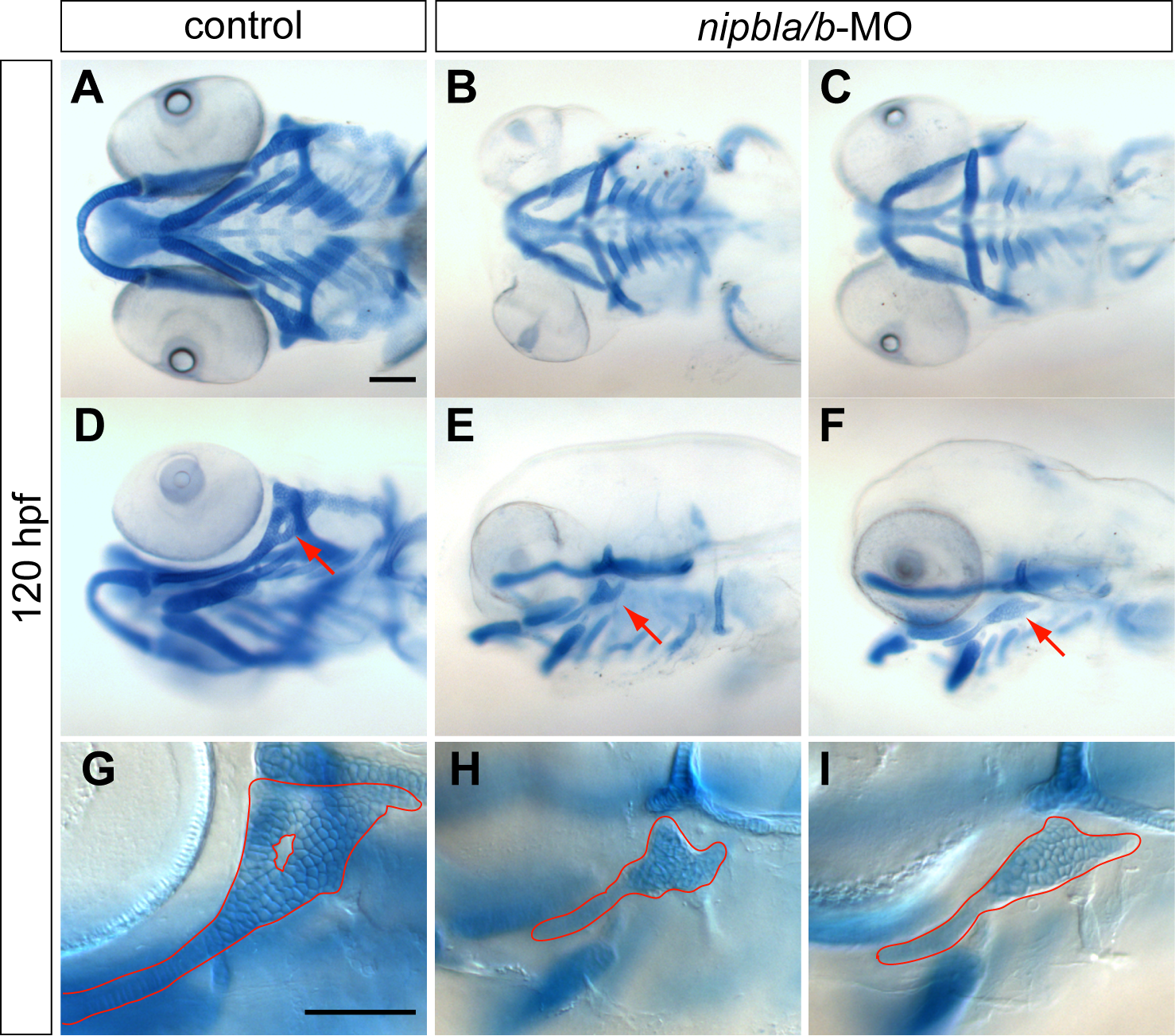

Supplement: Figure S5 — Defects in the larval craniofacial skeleton in nipbla/b-morphants. Craniofacial cartilages were stained with Alcian blue at 120 hpf. Controls (A, D, G) and two examples of nipbla/b-morphants (B, C, E, F, H, I) are shown in ventral (A–C), ventrolateral (D), and lateral view (E–I), anterior to the left. Specific reductions in hyosymplectic cartilages are indicated by arrows (D–F) and outlined at higher magnification (G–I). Scale bar: 100 µm. (TIF) [file pbio.1001181.s005.tif]
