# Supplementary material for: Multifactorial Origins of Heart and Gut Defects in nipbl-Deficient Zebrafish, a Model of Cornelia de Lange Syndrome
Source: PLoS Biol. 2011 Oct 25;9(10):e1001181. doi: 10.1371/journal.pbio.1001181 (PMC3201921; doi:10.1371/journal.pbio.1001181)
Supplement: Table S1 — Primers used for Q-PCR. (DOC) [file pbio.1001181.s012.doc]

**Table S1 Primers used for Q-PCR**

| genes | Sense | Antisense |
| --- | --- | --- |
| *sox17* | GCCAATGAACGCGTTTATGGT | CTCCTCAACGAATGGACGTT |
| *foxa2* | CTACGGAGAGCCTGAGTGTT | CCGTGTTGACATAGGACATGT |
| *sox32* | CTCAGCAAAATACTTGGCAAGA | GCTTGTTGCACTTTCGTCGA |
| *ef-1a* | CTGGTTCAAGGGATGGAAGA | GGTACAGTTCCAATACCTCCA |
| *rpl13a* | TCTGGAGGACTGTAAGAGGTATGC | AGACGCACAATCTTGAGAGCAG |
| *pou5f1* | AAACTTGTATGGCAAAATGTTCA | TGTCGACAAACACCCGTTCA |
| *cxcr4a* | GGCTTATTACGGACACATCGT | CGTTGGGAGAAAGATCCTCT |
| *cyc* | TCGTGTTCCCTAAGAAGTACA | CAGGATGCAGGAACACGACT |
| *ntl* | ACTGGATGAAAGCACCCGTA | AATGTGAAGCGATCTCAGTAG |
| *eve1* | ACAACTGAACTATATCCCAACT | GGTTCTGGAACCAGACCTG |
| *tbx16* | CATCACCAAACCTGGCAGAA | CTCCCACTTATCCTTGTTCCA |
| *myca* | GGCAGCGATTCAGAAGATGAAG | CCGTCTCGTGCCTTTTCTGT |
| *ascl1a* | GGGCTCATACGACCCTCTGA | TCCCAAGCGAGTGCTGATATTT |
| *ascl1b* | CCACATGGTTCGACAGATACGA | CAGCATGCAGCAAATCAAAGAC |
| *p53* | CCCATCCTCACAATCATCACT | CACGCACCTCAAAAGACCTC |
| *mdm2* | GGTGCAATTGAAAAGCCTGT | GACTCCTGGGTTCAGAGAAGG |
