# Supplementary material for: Multifactorial Origins of Heart and Gut Defects in nipbl-Deficient Zebrafish, a Model of Cornelia de Lange Syndrome
Source: PLoS Biol. 2011 Oct 25;9(10):e1001181. doi: 10.1371/journal.pbio.1001181 (PMC3201921; doi:10.1371/journal.pbio.1001181)
Supplement: Table S2 — Phenotypes of nipbl-morphants. (DOC) [file pbio.1001181.s013.doc]

**Table S2. Phenotypes of *nipbl*-morphants.**

|  |  | | uninjected | control * | *nipbla-*  *MO1* | *nipblb-*  *MO1* | MO1-mix * | MO2-mix * |
| --- | --- | --- | --- | --- | --- | --- | --- | --- |
| **morphology (%)** | | |  |  |  |  |  |  |
| 34 hpf | | n | *132* | *82* | *55* | *61* | *195* | *30* |
| blood circulation defects | | | 0.0 | 7.3 | 52.7 | 1.6 | 87.2 | 50.0 |
| pericardiac edema | | | 0.0 | 0.0 | 52.7 | 0.0 | 88.7 | 50.0 |
| tail dysmorphology | | | 0.0 | 0.0 | 49.1 | 0.0 | 57.9 | 10.0 |
|  | | |  |  |  |  |  |  |
| 52 hpf | | n | *nt ***** | *15* | *nt ***** | *nt ***** | *30* | *nt ***** |
| urogenital opening defects | | |  | 0.0 |  |  | 10.0 |  |
|  | | |  |  |  |  |  |  |
| **heart (%)** ** | | |  |  |  |  |  |  |
| 32 hpf | | n | *132* | *82* | *55* | *61* | *195* | *30* |
| normal |  | | 97.0 | 100.0 | 43.6 | 93.5 | 7.7 | 23.3 |
| type A | reduced | | 0.0 | 0.0 | 18.2 | 4.9 | 32.8 | 60.0 |
|  | no jog | | 1.5 | 0.0 | 14.5 | 1.6 | 25.1 | 13.4 |
|  | reversed | | 1.5 | 0.0 | 1.8 | 0.0 | 1.5 | 0.0 |
| type B | fused | | 0.0 | 0.0 | 16.4 | 0.0 | 17.5 | 3.3 |
|  | cardia bifida | | 0.0 | 0.0 | 5.5 | 0.0 | 15.4 | 0.0 |
|  |  | |  |  |  |  |  |  |
| 48 hpf | | n | *nt ***** | *10* | *nt ***** | *nt ***** | *25* | *nt ***** |
| normal |  | |  | 100.0 |  |  | 4.0 |  |
| type A | reduced | |  | 0.0 |  |  | 36.0 |  |
|  | no loop | |  | 0.0 |  |  | 32.0 |  |
|  | reversed | |  | 0.0 |  |  | 0.0 |  |
| type B | fused | |  | 0.0 |  |  | 20.0 |  |
|  | cardia bifida | |  | 0.0 |  |  | 8.0 |  |
|  |  | |  |  |  |  |  |  |
| **visceral organs (%)** *** | | |  |  |  |  |  |  |
| 52 hpf | | n | *13* | *21* | *nt ***** | *nt ***** | *96* | *30* |
| normal |  | | 100.0 | 95.2 |  |  | 2.2 | 6.7 |
| type I | reduced | | 0.0 | 4.8 |  |  | 39.6 | 70.0 |
|  | no loop | | 0.0 | 0.0 |  |  | 25.8 | 6.7 |
|  | reversed | | 0.0 | 0.0 |  |  | 1.0 | 3.3 |
| type II |  | | 0.0 | 0.0 |  |  | 11.9 | 10.0 |
| type III |  | | 0.0 | 0.0 |  |  | 19.5 | 3.3 |

Defects in morphology were observed at 32 and 52 hpf using brightfield microscopy. Heart (32 and 48 hpf) and visceral organ (52 hpf) phenotypes were examined by ISH, using *cmlc2* and *foxa3* probes, respectively.

* Control embryos were co-injected with 5mis-*nipbla*-MO1 and 5mis-*nipblb*-MO1. MO1-mix and MO2-mix refer to co-injection of *nipbla*-MO1/*nipblb*-MO1 and *nipbla*-MO2/*nipblb*-MO2, respectively. All MOs except *nipbla*-MO2 (1.5 ng) were used at 0.75 ng per embryo.

** Type A: Defects in heart tube jogging/looping. Type B: Defects in medial migration of cardiac precursors. The heart tube is not formed in type B embryos.

*** Type I: Defects in gut tube looping. Type II: Bifurcation of anterior gut tube accompanied by bilateral duplications of visceral organs. Type III: Significant reduction in endoderm cells expressing *foxa3*. Type I and II embryos also had small liver and pancreas.

**** not tested.
