# Supplementary material for: Multifactorial Origins of Heart and Gut Defects in nipbl-Deficient Zebrafish, a Model of Cornelia de Lange Syndrome
Source: PLoS Biol. 2011 Oct 25;9(10):e1001181. doi: 10.1371/journal.pbio.1001181 (PMC3201921; doi:10.1371/journal.pbio.1001181)
Supplement: Table S3 — 600 genes potentially down- or up-regulated in nipbla/b-morphants. (DOC) [file pbio.1001181.s014.doc]

**Table S3. 600 genes potentially down- or up-regulated in *nipbla/b*-morphants.**

| Transcripts showing reduced expression | | | | |
| --- | --- | --- | --- | --- |
| Rank | Affymetrix Features | Gene description | Gene symbol | Expression ratio (MO/uninject) |
| 1 | Dr.10283.1.A1_at | BCL2/adenovirus E1B interacting protein 3-like | bnip3l | 0.33 |
| 2 | Dr.8107.1.S1_at | SRY-box containing gene 17 | sox17 | 0.41 |
| 3 | Dr.8085.1.S1_at | frizzled-related protein | frzb | 0.51 |
| 4 | Dr.25133.1.S1_at | myeloid cell leukemia sequence 1b | mcl1b | 0.51 |
| 5 | Dr.483.1.S1_at | forkhead box A2 | foxa2 | 0.52 |
| 6 | Dr.8202.2.S1_a_at | paired-like homeodomain transcription factor 2a | pitx2a | 0.53 |
| 7 | Dr.2953.2.S1_at | sorting nexin 3 (zgc:123189) | snx3 | 0.53 |
| 8 | Dr.11260.1.A1_at | Transcribed locus | --- | 0.56 |
| 9 | Dr.4334.1.A1_at | hypothetical LOC560815 | LOC560815 | 0.56 |
| 10 | Dr.7824.1.S1_at | ras homolog gene family, member Ab | rhoab | 0.57 |
| 11 | Dr.1251.1.S1_at | degenerative spermatocyte homolog 1, lipid desaturase | degs1 | 0.57 |
| 12 | Dr.15862.1.S1_at | BCL2/adenovirus E1B interacting protein 3-like | bnip3l | 0.57 |
| 13 | Dr.10694.1.S1_at | actin, alpha, cardiac muscle 1 like | actc1l | 0.58 |
| 14 | Dr.12777.1.S1_at | protocadherin 10b | pcdh10b | 0.53 |
| 15 | Dr.25548.1.S1_at | heat shock protein 90kDa beta (grp94), member 1 | hsp90b1 | 0.59 |
| 16 | Dr.25683.1.S1_at | cathepsin L, 1 b | ctsl1b | 0.46 |
| 17 | Dr.5682.1.S1_at | zgc:56513 | zgc:56513 | 0.48 |
| 18 | Dr.85.1.S1_at | nucleobindin 2a | nucb2a | 0.61 |
| 19 | Dr.289.1.S1_a_at | goosecoid | gsc | 0.61 |
| 20 | Dr.7103.1.S1_at | inhibitor of DNA binding 3 | id3 | 0.62 |
| 21 | Dr.15434.1.S1_at | queuine tRNA-ribosyltransferase domain containing 1 | qtrtd1 | 0.63 |
| 22 | Dr.8723.1.S1_at | zgc:92903 | zgc:92903 | 0.56 |
| 23 | Dr.12060.1.A1_at | holocarboxylase synthetase (biotin-(proprionyl-Coenzyme A-carboxylase (ATP-hydrolysing)) ligase) | hlcs | 0.63 |
| 24 | Dr.19408.1.A1_at | Wu:fc12c11 | wu:fc12c11 | 0.63 |
| 25 | Dr.12375.1.S1_at | --- | --- | 0.65 |
| 26 | Dr.4495.2.S1_at | dystrophin | dmd | 0.65 |
| 27 | DrAffx.1.2.S1_at | bhikhari | bhik | 0.65 |
| 28 | Dr.6709.1.S1_at | hypothetical LOC565309 | LOC565309 | 0.65 |
| 29 | Dr.12716.1.A1_at | si:dkey-264g21.1 | si:dkey-264g21.1 | 0.65 |
| 30 | Dr.8135.1.S1_at | proteasome activator subunit 1 | psme1 | 0.65 |
| 31 | Dr.3936.1.A1_at | similar to CCCH zinc finger protein C3H-1 | zgc:162730 | 0.65 |
| 32 | Dr.10727.1.S1_at | sine oculis homeobox homolog 4.1 | six4.1 | 0.65 |
| 33 | Dr.450.1.S1_at | carbonic anhydrase | cahz | 0.66 |
| 34 | Dr.11690.1.S1_at | ntl-dependent gene 5 | ntd5 | 0.61 |
| 35 | Dr.25615.1.A1_at | wu:fb96e11 | wu:fb96e11 | 0.66 |
| 36 | Dr.24890.1.S1_at | hypothetical protein LOC100002805 | LOC100002805 | 0.66 |
| 37 | Dr.16048.1.S1_at | myelocytomatosis oncogene b | mycb | 0.66 |
| 38 | Dr.3305.1.S1_at | ubiquitin-conjugating enzyme E2G 1 (UBC7 homolog, yeast) | ube2g1 | 0.67 |
| 39 | Dr.5434.5.S1_a_at | glycoprotein M6Ab | gpm6ab | 0.62 |
| 40 | Dr.13289.1.A1_at | Transcribed locus | --- | 0.67 |
| 41 | Dr.12825.1.A1_at | sine oculis homeobox homolog 7 | six7 | 0.61 |
| 42 | Dr.21612.1.A1_at | wu:fc25f11 | wu:fc25f11 | 0.67 |
| 43 | Dr.12425.1.S1_at | zgc:92533 | zgc:92533 | 0.68 |
| 44 | Dr.5887.1.A1_at | zgc:153685 | zgc:153685 | 0.68 |
| 45 | Dr.7532.1.A1_at | Transcribed locus, strongly similar to XP_001344088.1 hypothetical protein [Danio rerio] | --- | 0.68 |
| 46 | Dr.4661.1.A1_at | hypothetical LOC559003 | LOC559003 | 0.68 |
| 47 | Dr.12.1.S2_at | platelet derived growth factor receptor alpha | pdgfra | 0.69 |
| 48 | Dr.17395.1.A1_at | --- | --- | 0.69 |
| 49 | Dr.5477.1.S1_at | prothymosin, alpha | ptma | 0.70 |
| 50 | Dr.1192.2.S1_at | zgc:153154 | zgc:153154 | 0.68 |
| 51 | Dr.18309.1.S1_at | T-box 24 | tbx24 | 0.69 |
| 52 | Dr.8181.1.S1_at | frizzled homolog 8b | fzd8b | 0.70 |
| 53 | Dr.2855.1.A1_a_at | similar to ATPase, H+ transporting, lysosomal 70kDa, V1 subunit A, like | LOC564804 | 0.68 |
| 54 | Dr.5753.1.S1_at | sonic hedgehog b | shhb | 0.66 |
| 55 | Dr.17108.1.S1_at | hypothetical protein LOC100000862 | LOC100000862 | 0.70 |
| 56 | Dr.25176.5.A1_at | hypothetical protein LOC796662 | LOC796662 | 0.70 |
| 57 | Dr.2795.1.A1_at | hypothetical protein LOC792183 | LOC792183 | 0.70 |
| 58 | Dr.18696.1.S1_at | hypothetical LOC566067 | LOC566067 | 0.71 |
| 59 | Dr.2575.1.A1_at | hypothetical LOC568995 | LOC568995 | 0.71 |
| 60 | Dr.8282.2.S1_a_at | iroquois homeobox protein 3a | irx3a | 0.71 |
| 61 | Dr.13178.1.A1_at | wu:fc17f11 | wu:fc17f11 | 0.71 |
| 62 | Dr.8120.1.S1_at | noggin 1 | nog1 | 0.71 |
| 63 | Dr.17014.1.S1_at | Purkinje cell protein 4 like 1 | pcp4l1 | 0.72 |
| 64 | Dr.20440.1.A1_at | Zgc:86889 | zgc:86889 | 0.72 |
| 65 | Dr.22150.2.A1_at | zgc:158314 | zgc:158314 | 0.72 |
| 66 | Dr.5107.1.A1_at | si:ch211-260p9.1 | si:ch211-260p9.1 | 0.72 |
| 67 | Dr.24241.1.S1_at | midkine-related growth factor b | mdkb | 0.64 |
| 68 | Dr.14535.1.S1_at | similar to LOC562179 protein | LOC100000271 | 0.71 |
| 69 | Dr.7882.1.A1_a_at | zgc:91941 | zgc:91941 | 0.72 |
| 70 | Dr.25285.1.S1_at | zgc:73223 | zgc:73223 | 0.72 |
| 71 | Dr.19254.1.A1_at | forkhead box C1b | foxc1b | 0.72 |
| 72 | Dr.6948.1.A1_at | zgc:152832 | zgc:152832 | 0.72 |
| 73 | Dr.9751.1.S1_at | flavin containing monooxygenase 5 | fmo5 | 0.68 |
| 74 | Dr.13384.1.S1_at | chordin | chd | 0.72 |
| 75 | Dr.3459.1.S1_at | Cd63 antigen | cd63 | 0.71 |
| 76 | Dr.6202.1.A1_at | zgc:91860 | zgc:91860 | 0.73 |
| 77 | Dr.25133.3.S1_at | hypothetical LOC561590 | LOC561590 | 0.73 |
| 78 | Dr.6295.1.S1_at | LIM domain only 4 | lmo4 | 0.73 |
| 79 | Dr.7506.1.A1_at | tubulin, alpha 8 like 2 | tuba8l2 | 0.73 |
| 80 | Dr.4868.1.A1_at | hypothetical LOC558116 | LOC558116 | 0.73 |
| 81 | Dr.2620.1.A1_at | hypothetical protein LOC553336 | LOC553336 | 0.73 |
| 82 | Dr.25566.1.S1_at | U1 small nuclear ribonucleoprotein polypeptide A | snrp70 | 0.73 |
| 83 | Dr.13872.1.A1_at | zgc:110269 | zgc:110269 | 0.73 |
| 84 | Dr.4258.1.A1_at | splicing factor, arginine/serine-rich 1 (splicing factor 2, alternate splicing factor) | sfrs1 | 0.73 |
| 85 | Dr.12366.1.S1_at | v-ets erythroblastosis virus E26 oncogene homolog 1a | ets1a | 0.74 |
| 86 | Dr.3562.1.A1_at | zgc:110308 | zgc:110308 | 0.74 |
| 87 | Dr.413.2.A1_at | hypothetical protein LOC792823 | LOC792823 | 0.74 |
| 88 | Dr.21888.1.S1_at | hypothetical protein LOC100005261 | LOC100005261 | 0.61 |
| 89 | Dr.12726.1.S1_at | Yes-relayed kinase | yrk | 0.74 |
| 90 | Dr.813.1.S1_at | acetyl-CoA acetyltransferase 2 | acat2 | 0.74 |
| 91 | Dr.8208.1.S1_at | lefty1 | lft1 | 0.74 |
| 92 | Dr.25166.1.S1_at | im:6892314 | im:6892314 | 0.74 |
| 93 | Dr.5926.1.A1_at | zgc:152968 | zgc:152968 | 0.72 |
| 94 | Dr.26384.1.A1_at | wu:fc48a01 | wu:fc48a01 | 0.73 |
| 95 | Dr.19965.1.S1_at | fibronectin 1 | fn1 | 0.74 |
| 96 | DrAffx.2.56.A1_at | cytochrome P450 CYP2AA3v1 | cyp2aa3v1 | 0.74 |
| 97 | Dr.6099.1.S1_at | zgc:77481 | zgc:77481 | 0.58 |
| 98 | Dr.14876.1.A1_at | Transcribed locus | --- | 0.74 |
| 99 | Dr.5286.1.S1_at | zgc:158642 | zgc:158642 | 0.73 |
| 100 | Dr.10723.1.S1_at | T-box 1, brain | tbr1 | 0.75 |
| 101 | Dr.2621.1.A1_at | ancient ubiquitous protein 1 | aup1 | 0.75 |
| 102 | DrAffx.1.45.S1_at | zinc finger-like gene 1 | znfl1 | 0.75 |
| 103 | Dr.1128.1.S1_at | carboxypeptidase N, polypeptide 1 | cpn1 | 0.75 |
| 104 | Dr.5072.1.A1_at | wu:fc13g09 | wu:fc13g09 | 0.75 |
| 105 | Dr.2654.1.A1_at | zgc:91808 | zgc:91808 | 0.75 |
| 106 | Dr.25243.1.S1_at | zgc:113200 | zgc:113200 | 0.75 |
| 107 | Dr.15827.1.A1_at | intraflagellar transport protein 52 | ift52 | 0.75 |
| 108 | Dr.4270.1.A1_at | zgc:110687 | zgc:110687 | 0.75 |
| 109 | Dr.14446.1.S1_at | Transcribed locus | --- | 0.75 |
| 110 | Dr.2954.1.S1_at | similar to N-acetylneuraminate pyruvate lyase (dihydrodipicolinate synthase) | LOC100002401 | 0.75 |
| 111 | Dr.25060.3.A1_at | wu:fb53f04 | wu:fb53f04 | 0.75 |
| 112 | Dr.681.1.A1_at | zgc:65909 | zgc:65909 | 0.65 |
| 113 | Dr.3521.1.A1_at | wu:fb51e06 | wu:fb51e06 | 0.75 |
| 114 | Dr.6314.1.S1_at | superoxide dismutase 2, mitochondrial | sod2 | 0.73 |
| 115 | Dr.12425.1.S1_x_at | zgc:92533 | zgc:92533 | 0.75 |
| 116 | Dr.3719.1.A1_at | zgc:86863 | zgc:86863 | 0.75 |
| 117 | Dr.24239.1.S1_at | novel protein containing a ChaC-like protein domain | CH211-244P18.4 | 0.76 |
| 118 | Dr.11452.1.A1_at | zgc:153958 | zgc:153958 | 0.76 |
| 119 | Dr.7870.1.A1_at | Transcribed locus | --- | 0.76 |
| 120 | Dr.5635.2.S1_at | hypothetical protein LOC100001201 | LOC10000120 | 0.76 |
| 121 | Dr.25140.2.S1_a_at | ictacalcin | icn | 0.76 |
| 122 | Dr.4384.1.A1_at | similar to Solute carrier family 39 (metal ion transporter), member 5 | LOC561758 | 0.76 |
| 123 | Dr.604.1.S2_at | notch homolog 2 | notch2 | 0.76 |
| 124 | Dr.21921.1.A1_at | zgc:103654 | zgc:103654 | 0.76 |
| 125 | Dr.11940.1.A1_at | Transcribed locus | --- | 0.75 |
| 126 | Dr.8928.1.S1_at | CCR4-NOT transcription complex, subunit 8 | cnot8 | 0.76 |
| 127 | Dr.16296.1.S1_x_at | hypothetical LOC565189 | LOC565189 | 0.76 |
| 128 | Dr.11030.1.A1_at | zgc:73144 | zgc:73144 | 0.75 |
| 129 | Dr.15796.1.S1_at | collagen XV alpha 1 | col15a1 | 0.76 |
| 130 | Dr.4543.1.S1_at | similar to fibulin-4 | LOC572703 | 0.76 |
| 131 | Dr.20010.3.S2_at | eukaryotic translation initiation factor 5A | eif5a | 0.76 |
| 132 | Dr.1307.1.S1_at | forkhead box A3 | foxa3 | 0.76 |
| 133 | Dr.12334.1.A1_at | Transcribed locus | --- | 0.76 |
| 134 | Dr.10389.1.A1_at | tetraspanin 18 | tspan18 | 0.63 |
| 135 | Dr.9411.2.A1_at | zgc:158179 | zgc:158179 | 0.77 |
| 136 | Dr.1202.1.S1_at | xx:sd49 | xx:sd49 | 0.77 |
| 137 | Dr.822.1.S2_at | chemokine (C-X-C motif) ligand 12a (stromal cell-derived factor 1) | cxcl12a | 0.77 |
| 138 | Dr.11718.1.S1_at | zgc:123333 | zgc:123333 | 0.77 |
| 139 | Dr.21036.1.S1_at | si:xx-51f19.2 | si:xx-51f19.2 | 0.77 |
| 140 | Dr.19936.1.S1_at | T-box 1 | tbx1 | 0.77 |
| 141 | Dr.20434.1.A1_at | --- | --- | 0.77 |
| 142 | Dr.11127.2.A1_at | Zgc:92899 | zgc:92899 | 0.77 |
| 143 | Dr.26329.1.A1_at | clathrin, heavy polypeptide (Hc) | cltc | 0.77 |
| 144 | Dr.2646.1.A1_at | wu:fb13b04 | wu:fb13b04 | 0.77 |
| 145 | Dr.8617.1.A1_at | id:ibd5023 | id:ibd5023 | 0.77 |
| 146 | Dr.12.1.S1_at | platelet derived growth factor receptor alpha | pdgfra | 0.77 |
| 147 | Dr.5962.1.A1_at | Wu:fb13d05 | wu:fb13d05 | 0.68 |
| 148 | Dr.7379.1.A1_at | selenoprotein W, 2b | sepw2b | 0.77 |
| 149 | Dr.18084.1.A1_at | Hypothetical protein LOC794032 | LOC794032 | 0.77 |
| 150 | Dr.16062.1.S1_at | Hypothetical protein LOC100002196 | LOC100002196 | 0.77 |
| 151 | Dr.4543.2.S1_at | crystallin, gamma M4 | crygm4 | 0.73 |
| 152 | Dr.11310.3.S1_x_at | similar to tubulin alpha | LOC573216 | 0.71 |
| 153 | Dr.17438.1.S1_at | similar to TRAF2 binding protein | LOC100006206 | 0.77 |
| 154 | Dr.17882.1.A1_at | nidogen 2 (osteonidogen) | nid2 | 0.76 |
| 155 | Dr.26514.1.A1_at | similar to Nitrogen fixation gene, yeast homolog 1 | LOC562714 | 0.77 |
| 156 | Dr.20777.1.S2_at | thymidine kinase 1, soluble | tk1 | 0.77 |
| 157 | Dr.5109.1.A1_at | zgc:92123 | zgc:92123 | 0.77 |
| 158 | Dr.16296.1.S1_a_at | hypothetical LOC565189 | LOC565189 | 0.77 |
| 159 | Dr.13119.2.A1_at | Transcribed locus | --- | 0.77 |
| 160 | Dr.198.1.S1_at | follistatin | fst | 0.77 |
| 161 | Dr.2727.1.A1_at | --- | --- | 0.77 |
| 162 | Dr.3494.1.A1_at | thioredoxin-like 2 | txnl2 | 0.77 |
| 163 | Dr.16874.1.S1_at | zgc:66135 | zgc:66135 | 0.76 |
| 164 | Dr.25696.1.S1_at | si:ch211-51m24.3 | si:ch211-51m24.3 | 0.77 |
| 165 | Dr.15156.1.A1_at | CDC42 effector protein (Rho GTPase binding) 4 | cdc42ep4 | 0.77 |
| 166 | Dr.3421.1.A1_at | similar to ribosome binding protein 1 homolog 180kDa | LOC567029 | 0.77 |
| 167 | Dr.20912.1.S1_at | activated leukocyte cell adhesion molecule | alcam | 0.77 |
| 168 | Dr.26138.1.A1_at | Hypothetical protein LOC100004639 | LOC100004639 | 0.77 |
| 169 | Dr.18880.1.A1_at | Transcribed locus | --- | 0.76 |
| 170 | Dr.1605.1.S1_at | serine (or cysteine) proteinase inhibitor, clade A (alpha-1 antiproteinase, antitrypsin), member 1, like | serpina1l | 0.69 |
| 171 | Dr.2045.1.A1_at | L-3-hydroxyacyl-Coenzyme A dehydrogenase, short chain | hadhsc | 0.77 |
| 172 | Dr.1385.1.A1_at | zgc:112318 | zgc:112318 | 0.67 |
| 173 | Dr.25938.1.A1_at | Transcribed locus | --- | 0.78 |
| 174 | Dr.3827.1.A1_at | hypothetical LOC557315 | LOC557315 | 0.78 |
| 175 | Dr.22308.1.A1_at | hypothetical LOC570856 | LOC570856 | 0.78 |
| 176 | Dr.4605.1.A1_at | wu:fc33h04 | wu:fc33h04 | 0.78 |
| 177 | DrAffx.1.74.S1_at | selenoprotein W, 2b | sepw2b | 0.69 |
| 178 | Dr.1280.1.A1_at | CCAAT/enhancer binding protein (C/EBP), delta | cebpd | 0.77 |
| 179 | Dr.7535.1.A1_at | wu:fp56f09 | wu:fp56f09 | 0.78 |
| 180 | DrAffx.1.80.S1_at | parvalbumin 4 | pvalb4 | 0.78 |
| 181 | Dr.18834.1.S1_at | zgc:92414 | zgc:92414 | 0.78 |
| 182 | Dr.16296.2.A1_x_at | hypothetical protein LOC795072 | LOC795072 | 0.78 |
| 183 | Dr.12817.1.A1_at | im:7144703 | im:7144703 | 0.67 |
| 184 | Dr.24292.5.S1_at | --- | --- | 0.78 |
| 185 | DrAffx.2.105.S1_at | Transcribed locus | --- | 0.78 |
| 186 | Dr.17653.1.S1_at | zgc:91957 | zgc:91957 | 0.78 |
| 187 | Dr.12386.1.S1_x_at | mesogenin 1 | msgn1 | 0.76 |
| 188 | Dr.12437.1.A1_at | zgc:65831 | zgc:65831 | 0.78 |
| 189 | Dr.12867.1.A1_at | Transcribed locus | --- | 0.78 |
| 190 | Dr.14073.1.A1_at | similar to EPB49 protein | LOC565367 | 0.78 |
| 191 | Dr.25657.1.A1_at | zgc:77455 | zgc:77455 | 0.78 |
| 192 | Dr.4314.1.A1_a_at | sb:cb560 | sb:cb560 | 0.67 |
| 193 | Dr.18696.2.A1_at | hypothetical protein LOC796839 | LOC796839 | 0.78 |
| 194 | Dr.1889.1.S1_at | transferrin-a | tfa | 0.71 |
| 195 | Dr.3536.1.A1_at | similar to Collagen alpha 1(XI) chain precursor | LOC565402 | 0.78 |
| 196 | Dr.9781.1.S1_at | sine oculis homeobox homolog 4.2 | six4.2 | 0.78 |
| 197 | Dr.11518.1.A1_at | propionyl Coenzyme A carboxylase, beta polypeptide | pccb | 0.78 |
| 198 | Dr.184.1.S1_at | similar to beta-2-microglobulin | LOC796027 | 0.75 |
| 199 | Dr.25559.1.S1_at | sb:cb166 | sb:cb166 | 0.78 |
| 200 | Dr.4975.1.A1_at | wu:fb99e11 | wu:fb99e11 | 0.78 |
| 201 | Dr.61.1.A1_at | chemokine (C-X-C motif) ligand 12b (stromal cell-derived factor 1) | cxcl12b | 0.78 |
| 202 | Dr.4314.1.A1_x_at | sb:cb560 | sb:cb560 | 0.72 |
| 203 | Dr.5488.2.A1_at | similar to Apoa4 protein | LOC570354 | 0.78 |
| 204 | Dr.17892.1.S1_at | notch-regulated ankyrin repeat protein a | nrarpa | 0.78 |
| 205 | Dr.7538.1.S1_at | zgc:56201 | zgc:56201 | 0.78 |
| 206 | Dr.12169.2.S1_at | similar to Nudix (nucleoside diphosphate linked moiety X)-type motif 9 | LOC100003366 | 0.78 |
| 207 | Dr.18177.1.A1_at | similar to MGC53542 protein | LOC565576 | 0.78 |
| 208 | Dr.6325.1.A1_at | hypothetical LOC569206 | LOC569206 | 0.78 |
| 209 | Dr.2596.3.A1_at | betaine-homocysteine methyltransferase | bhmt | 0.75 |
| 210 | Dr.17529.1.A1_at | Transcribed locus | --- | 0.78 |
| 211 | AFFX-Dr-acta1-M_at | actin, alpha 1, skeletal muscle | acta1 | 0.79 |
| 212 | Dr.104.1.A1_at | hypothetical LOC568480 | LOC568480 | 0.79 |
| 213 | Dr.5133.1.S1_at | hypothetical LOC569053 | LOC569053 | 0.79 |
| 214 | Dr.16880.1.S1_at | hypothetical LOC572532 | LOC572532 | 0.79 |
| 215 | Dr.12386.2.S1_a_at | mesogenin 1 | msgn1 | 0.79 |
| 216 | Dr.8121.1.S1_at | twist1 | twist1 | 0.75 |
| 217 | Dr.23700.1.A1_at | wu:fb55h10 | wu:fb55h10 | 0.79 |
| 218 | AFFX-Dr-J01636-3_at | --- | --- | 0.79 |
| 219 | Dr.158.1.S1_at | distal-less homeobox gene 4a | dlx4a | 0.79 |
| 220 | Dr.9448.1.A1_at | zgc:154048 | zgc:154048 | 0.79 |
| 221 | Dr.22705.1.A1_at | --- | --- | 0.79 |
| 222 | Dr.11780.1.A1_at | mitochondrial topoisomerase I | top1mt | 0.79 |
| 223 | Dr.2596.1.S1_a_at | betaine-homocysteine methyltransferase | bhmt | 0.69 |
| 224 | Dr.4289.1.A1_at | wu:fb79d05 | wu:fb79d05 | 0.79 |
| 225 | Dr.12715.1.S1_at | CTAGE family, member 5 | ctage5 | 0.79 |
| 226 | Dr.12138.2.A1_at | wu:fd07d06 | wu:fd07d06 | 0.79 |
| 227 | Dr.12439.4.A1_x_at | hypothetical LOC564145 | LOC564145 | 0.78 |
| 228 | Dr.7928.1.A1_at | zgc:112335 | zgc:112335 | 0.79 |
| 229 | Dr.1543.1.S1_at | DnaJ (Hsp40) homolog, subfamily C, member 3 | dnajc3 | 0.75 |
| 230 | Dr.6502.2.S1_at | neurological oncogenic ventral antigen protein | nova1a | 0.79 |
| 231 | Dr.74.1.S1_at | similar to Homeobox protein Hox-B1b (Hox-A1) | LOC799789 | 0.60 |
| 232 | Dr.3336.1.S1_at | myeloid ecotropic viral integration 1 | meis1 | 0.79 |
| 233 | Dr.24499.1.A1_at | Similar to family with sequence similarity 40, member A | LOC556392 | 0.79 |
| 234 | Dr.1192.1.S1_at | prostaglandin D2 synthase | ptgds | 0.79 |
| 235 | Dr.5544.2.S1_a_at | B lymphoma Mo-MLV insertion region 1 | bmi1 | 0.79 |
| 236 | Dr.9512.1.A1_at | influenza virus NS1A binding protein a | ivns1abpa | 0.79 |
| 237 | Dr.9564.1.A1_at | wu:fj88f05 | wu:fj88f05 | 0.79 |
| 238 | Dr.25527.1.A1_at | similar to alpha-2-macroglobulin-1 | LOC572080 | 0.79 |
| 239 | Dr.12559.1.S1_at | hypothetical LOC566329 | LOC566329 | 0.79 |
| 240 | Dr.11310.1.S1_at | similar to alpha-tubulin isotype M-alpha-2 | LOC573122 | 0.79 |
| 241 | Dr.694.1.A1_at | wu:fb13h06 | wu:fb13h06 | 0.79 |
| 242 | Dr.22569.1.S1_at | inositol 1,3,4-triphosphate 5/6 kinase | itpk1 | 0.71 |
| 243 | Dr.21087.1.A1_at | deoxyribonuclease I-like 3 | dnase1l3 | 0.79 |
| 244 | Dr.14930.1.A1_at | Transcribed locus | --- | 0.79 |
| 245 | Dr.17136.1.A1_at | Transcribed locus | --- | 0.79 |
| 246 | Dr.7768.1.A1_at | split hand/foot malformation (ectrodactyly) type 1 | shfm1 | 0.79 |
| 247 | Dr.4628.1.S1_at | ATPase, H+ transporting, lysosomal accessory protein 1 | atp6ap1 | 0.79 |
| 248 | Dr.21652.1.A1_at | zgc:77415 | zgc:77415 | 0.78 |
| 249 | Dr.26540.1.A1_at | wu:fe25c07 | wu:fe25c07 | 0.70 |
| 250 | Dr.17896.2.A1_at | notch-regulated ankyrin repeat protein b | nrarpb | 0.79 |
| 251 | Dr.8946.1.A1_at | Transcribed locus | --- | 0.79 |
| 252 | Dr.2267.1.A1_at | hypothetical protein LOC793723 | LOC793723 | 0.79 |
| 253 | Dr.18579.1.S1_at | laminin, gamma 1 | lamc1 | 0.79 |
| 254 | Dr.25140.1.S1_at | DnaJ (Hsp40) homolog, subfamily A, member 2 | dnaja2 | 0.79 |
| 255 | Dr.17731.2.S1_at | zgc:92242 | zgc:92242 | 0.79 |
| 256 | Dr.4495.1.S1_a_at | dystrophin /// similar to dystrophin | dmd | 0.79 |
| 257 | Dr.14521.1.S1_at | hypothetical protein LOC100005591 | LOC100005591 | 0.79 |
| 258 | Dr.9849.1.A1_at | zgc:77060 | zgc:77060 | 0.79 |
| 259 | Dr.5299.2.A1_at | sorting nexin 12 | snx12 | 0.79 |
| 260 | Dr.21009.1.S1_at | nitric oxide synthase 1 (neuronal) | nos1 | 0.79 |
| 261 | Dr.12383.2.S1_a_at | beta-carotene 15,15'-monooxygenase 1 | bcmo1 | 0.79 |
| 262 | Dr.4744.1.S1_a_at | --- | --- | 0.79 |
| 263 | Dr.2855.2.S1_at | similar to ATPase, H+ transporting, lysosomal 70kDa, V1 subunit A, like | LOC564804 | 0.79 |
| 264 | Dr.1192.1.S1_a_at | prostaglandin D2 synthase | ptgds | 0.80 |
| 265 | Dr.17366.1.A1_at | Hypothetical LOC562199 | LOC562199 | 0.80 |
| 266 | Dr.17653.2.S1_a_at | --- | --- | 0.80 |
| 267 | DrAffx.1.64.S1_at | NK1 transcription factor related 2-like,a | nkx1.2la | 0.80 |
| 268 | Dr.23457.1.S1_s_at | wu:fc18a02 | wu:fc18a02 | 0.76 |
| 269 | Dr.4579.1.A1_at | hypothetical protein LOC791811 | LOC791811 | 0.80 |
| 270 | Dr.9480.1.S1_at | zgc:55605 | zgc:55605 | 0.80 |
| 271 | Dr.555.1.S1_at | neurogenin 1 | neurog1 | 0.80 |
| 272 | Dr.16239.1.A1_at | Similar to lipin 1 | LOC792392 | 0.80 |
| 273 | Dr.26116.1.S1_at | heat shock 70kDa protein 5 (glucose-regulated protein) | hspa5 | 0.80 |
| 274 | Dr.20639.1.S1_at | zgc:56065 | zgc:56065 | 0.80 |
| 275 | Dr.13750.1.S1_at | --- | --- | 0.80 |
| 276 | Dr.8100.1.S1_at | GATA-binding protein 5 | gata5 | 0.80 |
| 277 | Dr.26209.1.A1_at | --- | --- | 0.80 |
| 278 | Dr.18797.1.A1_at | --- | --- | 0.80 |
| 279 | Dr.7568.1.A1_at | si:ch211-132p20.4 | si:ch211-132p20.4 | 0.80 |
| 280 | Dr.20884.1.A1_at | --- | --- | 0.79 |
| 281 | Dr.11828.1.A1_at | zgc:77806 | zgc:77806 | 0.80 |
| 282 | Dr.22795.1.A1_at | hypothetical protein LOC798421 | LOC798421 | 0.80 |
| 283 | Dr.1735.1.A1_at | chloride intracellular channel 1 | clic1 | 0.80 |
| 284 | Dr.17977.1.S1_at | zgc:56702 | zgc:56702 | 0.80 |
| 285 | Dr.3562.2.S1_at | zgc:110308 | zgc:110308 | 0.80 |
| 286 | Dr.7728.1.A1_at | wu:fi38a11 | wu:fi38a11 | 0.80 |
| 287 | Dr.486.1.S1_at | protein phosphatase 1, regulatory (inhibitor) subunit 14B | ppp1r14b | 0.80 |
| 288 | Dr.4874.1.S1_at | RAB1A, member RAS oncogene family | rab1a | 0.80 |
| 289 | Dr.3817.1.S1_at | protein disulfide isomerase associated 4 | pdia4 | 0.78 |
| 290 | Dr.21742.1.A1_at | wu:fc51a12 | wu:fc51a12 | 0.80 |
| 291 | Dr.21669.1.S1_at | wu:fj01d05 | wu:fj01d05 | 0.80 |
| 292 | Dr.5122.1.S2_at | zgc:77366 | zgc:77366 | 0.80 |
| 293 | Dr.13466.1.A1_at | vitronectin | vtn | 0.77 |
| 294 | Dr.14868.1.S1_at | zgc:103699 | zgc:103699 | 0.80 |
| 295 | Dr.4877.1.S1_at | zgc:55865 | zgc:55865 | 0.80 |
| 296 | Dr.16130.1.S1_at | alcohol dehydrogenase 8b | adh8b | 0.80 |
| 297 | Dr.17871.1.S1_at | --- | --- | 0.80 |
| 298 | Dr.11237.1.S1_at | zgc:112056 | zgc:112056 | 0.80 |
| 299 | Dr.26438.1.S1_at | notch-regulated ankyrin repeat protein a | nrarpa | 0.80 |
| 300 | Dr.17015.1.S1_at | hypothetical protein LOC791600 | LOC791600 | 0.80 |

| Transcripts showing increased expression | | | | |
| --- | --- | --- | --- | --- |
| Rank | Affymetrix Features | Gene description | Gene symbol | Expression ratio (MO/uninject) |
| 1 | Dr.20400.1.A1_at | nuclear transcription factor Y, alpha | nfya | 4.59 |
| 2 | Dr.14439.1.A1_at | zgc:92812 | zgc:92812 | 2.86 |
| 3 | Dr.1249.1.A1_at | transmembrane and ubiquitin-like domain containing 1 | tmub1 | 2.78 |
| 4 | Dr.4066.1.S1_at | zgc:56558 | zgc:56558 | 2.61 |
| 5 | Dr.7657.1.A1_at | ST3 beta-galactoside alpha-2,3-sialyltransferase 2 | st3gal2l | 2.25 |
| 6 | Dr.18117.2.S1_a_at | neogenin 1 | neo1 | 2.24 |
| 7 | Dr.6370.1.A1_at | hypothetical LOC561993 | MGC162592 | 1.97 |
| 8 | Dr.3081.1.A1_at | zgc:63749 | zgc:63749 | 1.95 |
| 9 | Dr.22910.1.A1_at | G elongation factor, mitochondrial 2 | gfm2 | 1.92 |
| 10 | Dr.1063.1.A1_at | THO complex 1 | thoc1 | 1.75 |
| 11 | Dr.22502.1.A1_at | E2F transcription factor 4 | e2f4 | 1.73 |
| 12 | Dr.8575.1.A1_at | zgc:123254 | zgc:123254 | 1.71 |
| 13 | Dr.4561.1.S1_at | very low density lipoprotein receptor | vldlr | 1.70 |
| 14 | Dr.91.1.A1_at | zgc:73187 | zgc:73187 | 1.67 |
| 15 | Dr.20582.1.S2_at | RNA binding protein with multiple splicing 2 | rbpms2 | 1.60 |
| 16 | Dr.15978.1.A1_at | similar to protein phosphatase 1, regulatory subunit 15B | LOC571470 | 1.60 |
| 17 | Dr.13604.1.S2_at | glycogenin 1 | gyg1 | 1.57 |
| 18 | Dr.11719.1.S1_at | roundabout homolog 1 | robo1 | 1.56 |
| 19 | Dr.23128.1.A1_at | --- | --- | 1.53 |
| 20 | Dr.13827.1.A1_at | zgc:153865 | zgc:153865 | 1.55 |
| 21 | Dr.7338.1.S1_at | influenza virus NS1A binding protein b | ivns1abpb | 1.52 |
| 22 | Dr.11480.1.S1_at | Rap2 interacting protein | rap2ip | 1.52 |
| 23 | Dr.25684.1.S1_at | homeodomain leucine zipper gene | homez | 1.54 |
| 24 | Dr.9268.1.A1_at | --- | --- | 1.61 |
| 25 | Dr.6236.1.A1_at | hypothetical LOC558551 | LOC558551 | 1.51 |
| 26 | Dr.18433.1.A1_at | zgc:153232 | zgc:153232 | 1.50 |
| 27 | Dr.17279.1.A1_at | --- | --- | 1.50 |
| 28 | Dr.24873.1.A1_at | similar to R3H domain containing 1 | LOC560062 | 1.50 |
| 29 | Dr.2527.1.A1_at | hypothetical LOC562718 | LOC562718 | 1.49 |
| 30 | Dr.25952.1.A1_s_at | very low density lipoprotein receptor | vldlr | 1.47 |
| 31 | Dr.5846.1.A1_at | activating transcription factor 4 (tax-responsive enhancer element B67) | atf4 | 1.47 |
| 32 | Dr.25898.1.A1_at | THAP7 protein | THAP7 | 1.46 |
| 33 | Dr.12590.1.S1_at | jagged 1a | jag1a | 1.46 |
| 34 | Dr.16690.1.A1_at | zgc:85976 | zgc:85976 | 1.46 |
| 35 | Dr.9471.1.A1_at | caprin family member 2 | caprin2 | 1.45 |
| 36 | Dr.19004.1.S1_at | Splicing factor proline/glutamine rich (polypyrimidine tract binding protein associated) | sfpq | 1.45 |
| 37 | Dr.7352.1.A1_at | ubiquitin-conjugating enzyme E2D 2 (UBC4/5 homolog, yeast) | ube2d2 | 1.45 |
| 38 | Dr.19766.1.A1_at | zgc:110030 | zgc:110030 | 1.44 |
| 39 | Dr.17518.1.S1_at | hypothetical LOC558435 | LOC558435 | 1.44 |
| 40 | Dr.7337.1.S1_at | solute carrier family 16 (monocarboxylic acid transporters), member 3 | slc16a3 | 1.43 |
| 41 | Dr.4813.1.A1_at | zgc:158798 | zgc:158798 | 1.43 |
| 42 | Dr.20345.1.S1_at | hypothetical protein FLJ12949-like (H. sapiens) | flj12949l | 1.43 |
| 43 | Dr.24663.1.S1_at | zgc:56565 | zgc:56565 | 1.42 |
| 44 | Dr.3932.1.S1_at | procollagen-proline, 2-oxoglutarate 4-dioxygenase (proline 4-hydroxylase), alpha polypeptide I | p4ha1 | 1.42 |
| 45 | Dr.20789.1.A1_at | v-erb-b2 erythroblastic leukemia viral oncogene homolog 2, neuro/glioblastoma derived oncogene homolog | erbb2 | 1.40 |
| 46 | Dr.2175.1.A1_at | ovary-expressed homeobox protein | hacp | 1.40 |
| 47 | Dr.26372.1.A1_at | --- | --- | 1.40 |
| 48 | Dr.22065.1.A1_at | wu:fd11b07 | wu:fd11b07 | 1.47 |
| 49 | Dr.13991.1.S1_at | SUB1 homolog (S. cerevisiae) | sub1 | 1.39 |
| 50 | Dr.20582.1.S1_at | RNA binding protein with multiple splicing 2 | rbpms2 | 1.45 |
| 51 | Dr.16580.1.A1_at | endothelial PAS domain protein 1 | epas1 | 1.39 |
| 52 | Dr.3926.1.S1_at | hippocampus abundant transcript 1b | hiat1b | 1.39 |
| 53 | Dr.15075.1.A1_at | zgc:152929 | zgc:152929 | 1.44 |
| 54 | Dr.23041.1.A1_at | calcium/calmodulin-dependent protein kinase (CaM kinase) II beta | camk2b | 1.39 |
| 55 | Dr.991.2.A1_s_at | geranylgeranyl diphosphate synthase 1 | ggps1 | 1.38 |
| 56 | Dr.8.1.S1_at | zgc:77429 | zgc:77429 | 1.37 |
| 57 | Dr.22945.1.S1_at | hypothetical protein LOC795535 | LOC795535 | 1.37 |
| 58 | Dr.20116.1.A1_at | zgc:158239 | zgc:158239 | 1.37 |
| 59 | Dr.11398.1.A1_at | zgc:64188 | zgc:64188 | 1.42 |
| 60 | Dr.14867.1.A1_at | neurexophilin 1 | nxph1 | 1.36 |
| 61 | Dr.26058.1.A1_at | Transcribed locus | --- | 1.36 |
| 62 | Dr.4309.1.A1_at | similar to Im:6902697 protein | LOC571625 | 1.36 |
| 63 | Dr.5510.1.A1_at | wu:fc09b09 | wu:fc09b09 | 1.36 |
| 64 | Dr.7345.1.S1_at | zgc:113424 | zgc:113424 | 1.36 |
| 65 | Dr.3415.1.A1_at | --- | --- | 1.36 |
| 66 | Dr.7320.1.A1_at | zgc:66481 | zgc:66481 | 1.36 |
| 67 | Dr.17464.1.A1_at | hypothetical protein LOC571260 | LOC571260 | 1.36 |
| 68 | Dr.13743.2.A1_a_at | similar to MGC79571 protein | LOC568127 | 1.51 |
| 69 | Dr.4813.2.A1_at | zgc:158798 | zgc:158798 | 1.35 |
| 70 | Dr.8280.1.S2_at | decapentaplegic and Vg-related 1, RNA binding protein | dvr1rbp | 1.35 |
| 71 | Dr.25573.1.A1_at | wu:fj80h11 | wu:fj80h11 | 1.35 |
| 72 | Dr.17233.1.A1_at | CCR4-NOT transcription complex, subunit 10 | cnot10 | 1.35 |
| 73 | Dr.15212.1.A1_at | zgc:92140 | zgc:92140 | 1.37 |
| 74 | Dr.15888.1.A1_at | zgc:91862 | zgc:91862 | 1.35 |
| 75 | Dr.13601.1.A1_at | similar to transcription factor IIIA | LOC558030 | 1.35 |
| 76 | Dr.13760.1.S1_at | similar to thymosin beta b | LOC796588 | 1.38 |
| 77 | Dr.1162.1.S1_at | zgc:73237 | zgc:73237 | 1.34 |
| 78 | Dr.13765.1.S1_at | forkhead box P2 | foxp2 | 1.34 |
| 79 | Dr.15885.1.A1_at | zgc:103540 | zgc:103540 | 1.35 |
| 80 | Dr.9243.1.A1_at | zgc:92434 | zgc:92434 | 1.34 |
| 81 | Dr.17137.1.S1_at | RAR-related orphan receptor A, paralog b | rorab | 1.33 |
| 82 | Dr.4989.1.S1_at | chromosome 10 open reading frame 119 (H. sapiens) | c10orf119 | 1.33 |
| 83 | Dr.1909.1.S1_at | hypothetical LOC560869 | LOC560869 | 1.33 |
| 84 | Dr.7378.1.S1_at | --- | --- | 1.37 |
| 85 | Dr.1848.1.A1_a_at | zgc:66298 | zgc:66298 | 1.33 |
| 86 | Dr.15165.2.A1_at | si:ch211-196f5.1 | si:ch211-196f5.1 | 1.33 |
| 87 | Dr.14346.2.A1_x_at | similar to COASTER | LOC100001768 | 1.35 |
| 88 | Dr.6818.1.S1_at | myelin protein zero | grb10 | 1.33 |
| 89 | Dr.16000.1.S1_at | Similar to alanine aminotransferase 2 | LOC799963 | 1.36 |
| 90 | Dr.13953.1.A1_at | zgc:153423 | zgc:153423 | 1.32 |
| 91 | Dr.20.1.S1_at | zgc:92564 | zgc:92564 | 1.41 |
| 92 | Dr.25322.2.S1_at | lin-7 homolog C (C. elegans) | lin7c | 1.32 |
| 93 | Dr.26457.1.S1_at | v-ral simian leukemia viral oncogene homolog A (ras related) | rala | 1.32 |
| 94 | Dr.399.1.A1_at | zgc:64154 | zgc:64154 | 1.32 |
| 95 | Dr.935.1.S1_at | vesicle-associated membrane protein, associated protein B and C | vapb | 1.32 |
| 96 | Dr.14490.1.A1_at | im:7162785 | im:7162785 | 1.32 |
| 97 | Dr.6767.1.A1_at | zgc:55843 | zgc:55843 | 1.31 |
| 98 | Dr.7324.1.A1_at | hypothetical LOC562677 | LOC562677 | 1.36 |
| 99 | Dr.17242.1.A1_at | zgc:76924 | zgc:76924 | 1.31 |
| 100 | Dr.26526.1.S1_at | zgc:110686 | zgc:110686 | 1.31 |
| 101 | Dr.10676.1.S1_at | CDP-diacylglycerol synthase (phosphatidate cytidylyltransferase) 2 | cds2 | 1.31 |
| 102 | Dr.4749.1.A1_at | zgc:92116 | zgc:92116 | 1.34 |
| 103 | Dr.5429.1.S1_at | si:dkey-15h8.11 | si:dkey-15h8.11 | 1.31 |
| 104 | Dr.14001.1.A1_at | zgc:103708 | zgc:103708 | 1.34 |
| 105 | Dr.841.1.A1_at | similar to Acheron | LOC565767 | 1.31 |
| 106 | Dr.25552.1.S1_at | zgc:55996 | zgc:55996 | 1.31 |
| 107 | Dr.21628.1.A1_at | wu:fc26d05 | wu:fc26d05 | 1.31 |
| 108 | Dr.3270.1.S1_at | actin related protein 2/3 complex, subunit 1A | arpc1a | 1.30 |
| 109 | Dr.12231.1.S1_at | sarcoma amplified sequence | sas | 1.30 |
| 110 | Dr.8402.1.S1_at | --- | --- | 1.30 |
| 111 | Dr.4350.1.A1_at | zgc:77004 | zgc:77004 | 1.30 |
| 112 | Dr.10451.1.S1_at | MKK4 | zMKK4 | 1.30 |
| 113 | Dr.5123.1.A1_at | zgc:92094 | zgc:92094 | 1.30 |
| 114 | Dr.1695.1.A1_at | similar to Im:7150924 protein | LOC795869 | 1.30 |
| 115 | Dr.6794.1.S1_at | zgc:63994 | zgc:63994 | 1.33 |
| 116 | Dr.15438.1.A1_at | tuberous sclerosis 1a | tsc1a | 1.30 |
| 117 | Dr.5335.1.S2_at | pl10 | pl10 | 1.29 |
| 118 | Dr.13601.2.S1_at | similar to transcription factor IIIA | LOC558030 | 1.31 |
| 119 | Dr.3559.1.A1_at | tetraspanin 7b | tspan7b | 1.29 |
| 120 | Dr.5335.1.S1_at | pl10 | pl10 | 1.31 |
| 121 | Dr.7638.1.S2_at | calmodulin 3a (phosphorylase kinase, delta) | calm3a | 1.29 |
| 122 | Dr.1357.1.S1_at | chaperonin containing TCP1, subunit 8 (theta) | cct8 | 1.29 |
| 123 | Dr.2440.1.A1_at | epiphycan | epyc | 1.29 |
| 124 | Dr.6727.1.A1_at | similar to R3H domain containing 1 | LOC560062 | 1.29 |
| 125 | Dr.20479.1.S1_at | zgc:158780 | zgc:158780 | 1.29 |
| 126 | Dr.15230.1.A1_at | Transcribed locus | --- | 1.29 |
| 127 | Dr.24969.1.S1_at | alkaline phosphatase | alp | 1.29 |
| 128 | Dr.17075.1.A1_at | hypothetical protein LOC100000821 | LOC100000821 | 1.29 |
| 129 | Dr.22410.1.A1_at | bromodomain containing 3b | brd3b | 1.29 |
| 130 | Dr.20143.1.S1_at | hairy-related 3 | her3 | 1.29 |
| 131 | Dr.7605.1.A1_at | wu:fj36c11 | wu:fj36c11 | 1.29 |
| 132 | Dr.25852.1.A1_at | lin-7 homolog C (C. elegans) | lin7c | 1.29 |
| 133 | Dr.15205.1.A1_at | Transcribed locus | --- | 1.28 |
| 134 | Dr.18079.1.S1_at | Kinesin-associated protein 3 | kifap3 | 1.28 |
| 135 | Dr.7274.1.S1_at | 6-phosphofructo-2-kinase/fructose-2,6-biphosphatase 1 | pfkfb1 | 1.28 |
| 136 | Dr.5037.1.A1_at | si:dkey-252h13.6 | si:dkey-252h13.6 | 1.28 |
| 137 | Dr.3273.1.A1_at | wu:fc57a01 | wu:fc57a01 | 1.28 |
| 138 | Dr.12444.1.A1_at | --- | --- | 1.28 |
| 139 | Dr.8325.1.S1_at | mix-type homeobox gene 2 | mxtx2 | 1.31 |
| 140 | Dr.26316.1.A1_at | Transcribed locus | --- | 1.28 |
| 141 | Dr.19756.1.A1_at | zgc:153537 | zgc:153537 | 1.28 |
| 142 | Dr.10517.1.A1_at | wu:fd15a06 | wu:fd15a06 | 1.28 |
| 143 | Dr.12209.1.S1_at | similar to alanine aminotransferase 2 | LOC799963 | 1.30 |
| 144 | Dr.2410.1.A1_at | similar to Branched chain keto acid dehydrogenase E1, beta polypeptide (maple syrup urine disease) | LOC569980 | 1.28 |
| 145 | Dr.6288.1.S1_at | zgc:56517 | zgc:56517 | 1.28 |
| 146 | Dr.11713.1.S1_at | zgc:77421 | zgc:77421 | 1.28 |
| 147 | Dr.14058.1.A1_at | hypothetical LOC563972 | LOC563972 | 1.29 |
| 148 | Dr.20167.5.S1_at | --- | --- | 1.38 |
| 149 | Dr.2525.1.A1_at | Hypothetical protein LOC100003193 | LOC100003193 | 1.27 |
| 150 | Dr.12880.1.A1_at | --- | --- | 1.27 |
| 151 | Dr.26427.1.A1_at | zgc:158157 | zgc:158157 | 1.27 |
| 152 | Dr.20.3.S1_x_at | zgc:92564 | zgc:92564 | 1.27 |
| 153 | Dr.14785.2.A1_at | Transcribed locus, weakly similar to NP_034360.2 1 isoform 1 [Mus musculus] | --- | 1.27 |
| 154 | Dr.2503.1.S1_at | kinectin 1 | ktn1 | 1.29 |
| 155 | Dr.5306.1.S1_at | NOL1/NOP2/Sun domain family, member 2 | nsun2 | 1.27 |
| 156 | Dr.5790.1.A1_at | wu:fb95h01 | wu:fb95h01 | 1.27 |
| 157 | Dr.20010.8.A1_at | SRY-box containing gene 3 | sox3 | 1.27 |
| 158 | Dr.17647.1.A1_at | wu:fc16b12 | wu:fc16b12 | 1.32 |
| 159 | Dr.3849.1.A1_at | histone deacetylase 8 | hdac8 | 1.27 |
| 160 | Dr.15723.1.S1_at | dachshund b | dachb | 1.27 |
| 161 | Dr.21550.1.S1_at | dihydropyrimidinase-like 5a | dpysl5a | 1.27 |
| 162 | Dr.20567.1.S1_at | zgc:92575 | zgc:92575 | 1.27 |
| 163 | Dr.9174.1.S1_at | hypothetical protein LOC100000209 | LOC100000209 | 1.27 |
| 164 | Dr.22250.1.A1_at | zgc:66442 | zgc:66442 | 1.27 |
| 165 | Dr.2490.1.A1_at | wu:fj66a01 | wu:fj66a01 | 1.26 |
| 166 | Dr.5435.1.S1_at | eukaryotic translation initiation factor 5 | eif5 | 1.26 |
| 167 | Dr.12565.1.A1_at | hypothetical protein LOC100005890 | LOC100005890 | 1.26 |
| 168 | Dr.14942.1.S1_at | wu:fk23f11 | wu:fk23f11 | 1.26 |
| 169 | Dr.18389.1.S1_at | YTH domain family 3 | ythdf3 | 1.26 |
| 170 | Dr.716.1.A1_at | zgc:154168 | zgc:154168 | 1.26 |
| 171 | Dr.12804.1.S1_at | zgc:153708 | zgc:153708 | 1.26 |
| 172 | Dr.6127.1.A1_at | similar to Probable tyrosyl-tRNA synthetase, mitochondrial precursor (Tyrosine--tRNA ligase) (TyrRS) | LOC556702 | 1.26 |
| 173 | Dr.14493.1.A1_at | zgc:158624 | zgc:158624 | 1.26 |
| 174 | Dr.13763.1.S1_at | zgc:103459 | zgc:103459 | 1.26 |
| 175 | Dr.8412.1.A1_at | zgc:158804 | zgc:158804 | 1.26 |
| 176 | Dr.25151.1.S1_at | geranylgeranyl diphosphate synthase 1 | ggps1 | 1.26 |
| 177 | Dr.12596.1.S1_at | claudin c | cldnc | 1.28 |
| 178 | Dr.7973.1.S1_at | solute carrier family 35, member C2 | slc35c2 | 1.26 |
| 179 | Dr.11983.1.A1_at | zgc:152769 | zgc:152769 | 1.25 |
| 180 | Dr.17763.1.A1_at | Transcribed locus | --- | 1.26 |
| 181 | Dr.749.1.S1_at | t-complex polypeptide 1 | tcp1 | 1.25 |
| 182 | Dr.5307.1.S1_at | solute carrier family 20, member 1b | slc20a1b | 1.25 |
| 183 | Dr.13098.1.S1_at | Transcribed locus | --- | 1.25 |
| 184 | Dr.17269.1.A1_at | zgc:113886 | zgc:113886 | 1.27 |
| 185 | Dr.5660.1.S1_at | cysteine-rich protein 2 | crip2 | 1.36 |
| 186 | Dr.7225.1.S1_at | MOB1, Mps One Binder kinase activator-like 1A (yeast) | mobkl1a | 1.25 |
| 187 | Dr.24758.1.A1_at | zgc:153426 | zgc:153426 | 1.28 |
| 188 | Dr.9538.1.S1_at | zgc:55283 | zgc:55283 | 1.25 |
| 189 | Dr.17542.1.A1_at | similar to OTTXETP00000002159 | LOC556544 | 1.25 |
| 190 | Dr.7639.1.A1_at | hypothetical LOC567234 | LOC567234 | 1.32 |
| 191 | Dr.17248.1.A1_at | wu:fi76b10 | wu:fi76b10 | 1.25 |
| 192 | Dr.3473.1.S1_at | FK506 binding protein 4 | fkbp4 | 1.25 |
| 193 | Dr.111.1.A1_at | similar to Homeodomain leucine zipper gene | LOC572921 | 1.25 |
| 194 | Dr.8653.1.A1_at | hypothetical protein LOC791482 | LOC791482 | 1.25 |
| 195 | Dr.15313.1.A1_at | zgc:85662 | zgc:85662 | 1.25 |
| 196 | Dr.11138.1.A1_at | similar to podocalyxin-like protein | LOC563754 | 1.25 |
| 197 | Dr.2812.1.A1_at | WD repeat domain, phosphoinositide interacting 2 | wipi2 | 1.25 |
| 198 | Dr.23747.1.A1_at | wu:fa18c08 | wu:fa18c08 | 1.25 |
| 199 | Dr.16885.1.A1_at | zgc:110661 | zgc:110661 | 1.25 |
| 200 | Dr.18418.1.S1_at | zgc:66474 | zgc:66474 | 1.25 |
| 201 | Dr.9498.1.A1_at | wu:fj82d10 | wu:fj82d10 | 1.25 |
| 202 | Dr.12556.1.A1_at | zgc:123166 | zgc:123166 | 1.25 |
| 203 | Dr.17348.1.A1_at | hypothetical protein LOC100000599 | LOC100000599 | 1.24 |
| 204 | Dr.4980.1.S1_at | glutamate-ammonia ligase (glutamine synthase) b | glulb | 1.24 |
| 205 | Dr.7710.1.A1_at | zgc:92106 | zgc:92106 | 1.26 |
| 206 | Dr.24954.1.A1_at | heterogeneous nuclear ribonucleoprotein A0 | hnrpa0 | 1.24 |
| 207 | Dr.10065.1.A1_at | zgc:114139 | zgc:114139 | 1.24 |
| 208 | Dr.20197.1.A1_at | hypothetical protein LOC794007 | LOC794007 | 1.25 |
| 209 | Dr.9165.1.S1_at | zgc:55843 | zgc:55843 | 1.24 |
| 210 | Dr.11409.1.A1_at | zgc:113420 | zgc:113420 | 1.24 |
| 211 | Dr.4149.1.A1_at | hypothetical LOC566053 | LOC566053 | 1.24 |
| 212 | Dr.1524.1.S1_at | tyrosine 3-monooxygenase/tryptophan 5-monooxygenase activation protein, eta polypeptide | ywhah | 1.24 |
| 213 | Dr.14943.1.A1_at | Glyceronephosphate O-acyltransferase | gnpat | 1.24 |
| 214 | Dr.20459.1.S1_at | zgc:56280 | zgc:56280 | 1.33 |
| 215 | Dr.18766.1.A1_at | --- | --- | 1.24 |
| 216 | Dr.10424.2.S1_at | zgc:110182 | zgc:110182 | 1.24 |
| 217 | Dr.526.1.A1_at | acyl-CoA synthetase short-chain family member 2 | acss2 | 1.23 |
| 218 | Dr.16036.1.A1_at | zgc:114169 | zgc:114169 | 1.23 |
| 219 | Dr.16544.1.S1_at | zgc:153675 | zgc:153675 | 1.23 |
| 220 | Dr.20167.3.S1_at | --- | --- | 1.23 |
| 221 | Dr.3520.1.S1_at | germ cell-less homolog 1 (Drosophila) | gmcl1 | 1.23 |
| 222 | Dr.18883.1.A1_at | zgc:103754 | zgc:103754 | 1.23 |
| 223 | Dr.2353.1.S1_at | aconitase 2, mitochondrial | aco2 | 1.23 |
| 224 | Dr.9925.1.S1_at | zgc:73359 | zgc:73359 | 1.33 |
| 225 | Dr.1225.1.A1_at | LIM domain kinase 2 | limk2 | 1.23 |
| 226 | Dr.15648.1.A1_at | zgc:113258 | zgc:113258 | 1.23 |
| 227 | Dr.15857.2.S1_at | cysteine and glycine-rich protein 1 | csrp1 | 1.23 |
| 228 | Dr.5980.2.A1_at | similar to synaptic vesicle protein 2B | LOC556961 | 1.23 |
| 229 | Dr.17327.1.S1_at | zgc:56223 | zgc:56223 | 1.23 |
| 230 | Dr.1848.2.S1_at | zgc:66298 | zgc:66298 | 1.23 |
| 231 | Dr.5719.1.A1_at | claudin d | cldnd | 1.26 |
| 232 | Dr.17841.3.A1_at | zgc:114109 | zgc:114109 | 1.23 |
| 233 | Dr.5112.1.S3_at | SRY-box containing gene 11b | sox11b | 1.23 |
| 234 | Dr.991.1.S1_at | Geranylgeranyl diphosphate synthase 1 | ggps1 | 1.23 |
| 235 | Dr.13589.1.A1_at | zgc:110231 | zgc:110231 | 1.23 |
| 236 | Dr.18076.1.A1_at | zgc:92655 | zgc:92655 | 1.23 |
| 237 | Dr.6347.1.A1_at | de-etiolated homolog 1 (Arabidopsis) | det1 | 1.23 |
| 238 | Dr.14572.1.A1_at | Transcribed locus | --- | 1.23 |
| 239 | Dr.12945.1.A1_at | Similar to nuclear factor erythroid derived 2-like 1 | LOC794474 | 1.23 |
| 240 | Dr.16490.1.A1_at | si:ch211-284e13.1 | si:ch211-284e13.1 | 1.23 |
| 241 | Dr.13097.1.S1_at | 6-phosphofructo-2-kinase/fructose-2,6-biphosphatase 4 | pfkfb4 | 1.23 |
| 242 | Dr.11167.1.A1_at | Transcribed locus | --- | 1.23 |
| 243 | Dr.6766.1.A1_at | zgc:100952 | zgc:100952 | 1.23 |
| 244 | Dr.5123.2.S1_at | zgc:92094 | zgc:92094 | 1.24 |
| 245 | Dr.17654.1.A1_at | Similar to Peroxisomal coenzyme A diphosphatase NUDT7 (Nucleoside diphosphate-linked moiety X motif 7) (Nudix motif 7) | LOC564657 | 1.22 |
| 246 | Dr.15773.1.S1_at | zgc:153343 | zgc:153343 | 1.22 |
| 247 | Dr.14895.1.A1_at | hypothetical protein LOC100002469 | LOC100002469 | 1.22 |
| 248 | Dr.3736.1.A1_at | hypothetical LOC564572 | LOC564572 | 1.22 |
| 249 | Dr.2392.1.A1_at | NADH dehydrogenase (ubiquinone) Fe-S protein 8, (NADH-coenzyme Q reductase) | ndufs8 | 1.22 |
| 250 | Dr.17747.1.S1_at | sciellin | scel | 1.25 |
| 251 | Dr.2665.1.A1_at | hypothetical LOC572128 | LOC572128 | 1.22 |
| 252 | Dr.19046.1.A1_at | hypothetical protein LOC100001871 | LOC100001871 | 1.22 |
| 253 | Dr.13244.1.A1_at | zgc:152987 | zgc:152987 | 1.22 |
| 254 | Dr.24922.2.S1_at | zgc:103420 | zgc:103420 | 1.22 |
| 255 | Dr.14075.1.A1_at | si:ch211-63o20.5 | si:ch211-63o20.5 | 1.22 |
| 256 | Dr.15413.1.A1_at | Transcribed locus | --- | 1.22 |
| 257 | Dr.1691.14.S1_at | sb:cb8 | sb:cb8 | 1.22 |
| 258 | Dr.4322.1.A1_at | hypothetical LOC555702 | LOC555702 | 1.22 |
| 259 | Dr.12803.1.A1_at | --- | --- | 1.22 |
| 260 | Dr.13169.1.S1_at | receptor interacting protein kinase 5 | ripk5 | 1.22 |
| 261 | Dr.6027.1.A1_at | KDEL (Lys-Asp-Glu-Leu) endoplasmic reticulum protein retention receptor 3 | kdelr3 | 1.22 |
| 262 | Dr.4551.1.A1_at | wu:fc10a09 | wu:fc10a09 | 1.22 |
| 263 | Dr.13180.1.S1_at | zgc:56174 | zgc:56174 | 1.22 |
| 264 | Dr.13695.1.S1_at | similar to Gastrula zinc finger protein XLCGF57.1 | LOC100005923 | 1.22 |
| 265 | Dr.4330.1.A1_at | zgc:77793 | zgc:77793 | 1.22 |
| 266 | Dr.21282.1.A1_at | hypothetical protein LOC100000515 | LOC100000515 | 1.23 |
| 267 | Dr.3725.1.A1_at | zgc:101649 | zgc:101649 | 1.27 |
| 268 | Dr.12572.1.S1_at | v-mos Moloney murine sarcoma viral oncogene homolog | mos | 1.22 |
| 269 | Dr.9270.1.A1_at | zgc:110800 | zgc:110800 | 1.22 |
| 270 | Dr.25442.1.A1_at | elongation of very long chain fatty acids (FEN1/Elo2, SUR4/Elo3, yeast)-like 4 | elovl4 | 1.22 |
| 271 | Dr.5905.1.S1_at | zgc:56589 | zgc:56589 | 1.22 |
| 272 | Dr.26024.1.A1_at | hypothetical protein LOC100007020 | LOC100007020 | 1.25 |
| 273 | Dr.25672.1.A1_at | importin 9 | ipo9 | 1.22 |
| 274 | Dr.22039.1.A1_at | wu:fd07g01 | wu:fd07g01 | 1.22 |
| 275 | Dr.2619.1.S2_at | RNA binding motif protein, X-linked | rbmx | 1.22 |
| 276 | Dr.11528.1.A1_at | hypothetical protein LOC799498 | LOC799498 | 1.22 |
| 277 | Dr.15982.1.A1_at | Transcribed locus | --- | 1.22 |
| 278 | Dr.17248.2.S1_at | wu:fi76b10 | wu:fi76b10 | 1.22 |
| 279 | Dr.2475.1.A1_at | similar to phosphoinositide-3-kinase, class 2, alpha polypeptide | LOC571356 | 1.25 |
| 280 | Dr.18796.1.S1_at | zgc:110682 | zgc:110682 | 1.22 |
| 281 | Dr.25322.1.S1_at | Lin-7 homolog C (C. elegans) | lin7c | 1.26 |
| 282 | Dr.11829.1.A1_at | phosphotidylinositol transfer protein, beta | pitpnb | 1.22 |
| 283 | Dr.12319.1.A1_at | similar to phosphoinositide-3-kinase, class 2, alpha polypeptide | LOC571356 | 1.22 |
| 284 | Dr.10447.1.S1_at | ferrochelatase | fech | 1.22 |
| 285 | Dr.11791.1.A1_at | zgc:154102 | zgc:154102 | 1.22 |
| 286 | Dr.9746.5.A1_at | zgc:158601 | zgc:158601 | 1.22 |
| 287 | Dr.24200.1.S1_at | zgc:56049 | zgc:56049 | 1.22 |
| 288 | Dr.18175.2.S1_at | Transcribed locus | --- | 1.21 |
| 289 | Dr.23449.1.S1_at | hypothetical LOC564344 | LOC564344 | 1.21 |
| 290 | Dr.26338.1.A1_at | wu:fe01h05 | wu:fe01h05 | 1.21 |
| 291 | Dr.9238.1.A1_at | LATS, large tumor suppressor, homolog 1 (Drosophila) | lats1 | 1.21 |
| 292 | Dr.3168.2.A1_a_at | snurportin 1 | snupn | 1.21 |
| 293 | Dr.8687.1.S1_at | exportin 4 | xpo4 | 1.21 |
| 294 | Dr.3907.1.S1_at | wu:fc58e12 | wu:fc58e12 | 1.21 |
| 295 | Dr.22919.1.A1_at | wu:fc17g04 | wu:fc17g04 | 1.21 |
| 296 | Dr.479.2.A1_a_at | Wu:fb73a07 | wu:fb73a07 | 1.21 |
| 297 | Dr.199.1.A1_at | ELL associated factor 2 | eaf2 | 1.22 |
| 298 | Dr.18987.1.S1_at | Similar to autophagy protein 9 | LOC566801 | 1.21 |
| 299 | Dr.381.1.A1_at | --- | --- | 1.21 |
| 300 | Dr.16816.1.S1_at | si:dkey-34f16.5 | si:dkey-34f16.5 | 1.21 |
